# The Asp1 pyrophosphatase from *S. pombe* hosts a [2Fe-2S]^2+^ cluster in vivo

**DOI:** 10.1007/s00775-020-01840-w

**Published:** 2021-02-05

**Authors:** Hannah Rosenbach, Eva Walla, George E. Cutsail, James A. Birrell, Marina Pascual-Ortiz, Serena DeBeer, Ursula Fleig, Ingrid Span

**Affiliations:** 1 Institut Für Physikalische Biologie, Heinrich-Heine-Universität Düsseldorf, Universitätsstr. 1, 40225 Düsseldorf, Germany; 2 Lehrstuhl Für Funktionelle Genomforschung Der Mikroorganismen, Heinrich-Heine-Universität Düsseldorf, Universitätsstr. 1, 40225 Düsseldorf, Germany; 3 Max Planck Institute for Chemical Energy Conversion, Stiftstr. 34-36, 45470 Mülheim an der Ruhr, Germany; 4 Department of Biomedical Sciences, Faculty of Health Sciences, Universidad Cardenal Herrera, CEU Universities, 46113 Valencia, Spain

**Keywords:** PPIP5K/Vip1, *Schizosaccharomyces pombe*, Inositol phosphate metabolism, Iron–sulfur cluster, Pyrophosphatase, X-ray absorption spectroscopy

## Abstract

The *Schizosaccharomyces pombe* Asp1 protein is a bifunctional kinase/pyrophosphatase that belongs to the highly conserved eukaryotic diphosphoinositol pentakisphosphate kinase PPIP5K/Vip1 family. The N-terminal Asp1 kinase domain generates specific high-energy inositol pyrophosphate (IPP) molecules, which are hydrolyzed by the C-terminal Asp1 pyrophosphatase domain (Asp1^365−920^). Thus, Asp1 activities regulate the intracellular level of a specific class of IPP molecules, which control a wide number of biological processes ranging from cell morphogenesis to chromosome transmission. Recently, it was shown that chemical reconstitution of Asp1^371−920^ leads to the formation of a [2Fe-2S] cluster; however, the biological relevance of the cofactor remained under debate. In this study, we provide evidence for the presence of the Fe–S cluster in Asp1^365−920^ inside the cell. However, we show that the Fe–S cluster does not influence Asp1 pyrophosphatase activity in vitro or in vivo. Characterization of the as-isolated protein by electronic absorption spectroscopy, mass spectrometry, and X-ray absorption spectroscopy is consistent with the presence of a [2Fe-2S]^2+^ cluster in the enzyme. Furthermore, we have identified the cysteine ligands of the cluster. Overall, our work reveals that Asp1 contains an Fe–S cluster in vivo that is not involved in its pyrophosphatase activity.

**Supplementary Information:**

The online version contains supplementary material available at 10.1007/s00775-020-01840-w.

## Introduction

Iron–sulfur (Fe–S) clusters are ancient and versatile cofactors that are ubiquitously found in all organisms. Fe–S clusters associated with proteins are essential for numerous biological processes, including electron transfer, substrate binding and activation, redox catalysis, sensing of iron and oxygen, DNA replication and repair, regulation of gene expression, tRNA modification, and genome instability [1–4]. Fe–S clusters occur in nature in different shapes and nuclearities, and the most common types are the rhombic [2Fe-2S] cluster and the cubic [4Fe-4S] cluster [2]. Most Fe–S clusters are bound to the protein backbone by cysteine residues. In many Fe–S proteins, the cysteine ligands that bind the cofactor are arranged in characteristic cysteine pattern [5]. Despite the constant discovery of novel ligation patterns, it remains challenging to identify Fe–S-containing proteins purely based on sequence analysis. Another challenge in identifying protein-bound Fe–S cofactors is their sensitivity to oxygen [6–9]. Most Fe–S clusters degrade rapidly in the presence of atmospheric oxygen; thus, numerous native Fe–S proteins are isolated in the absence of the cofactor [10–12].

**Fig. 1:**
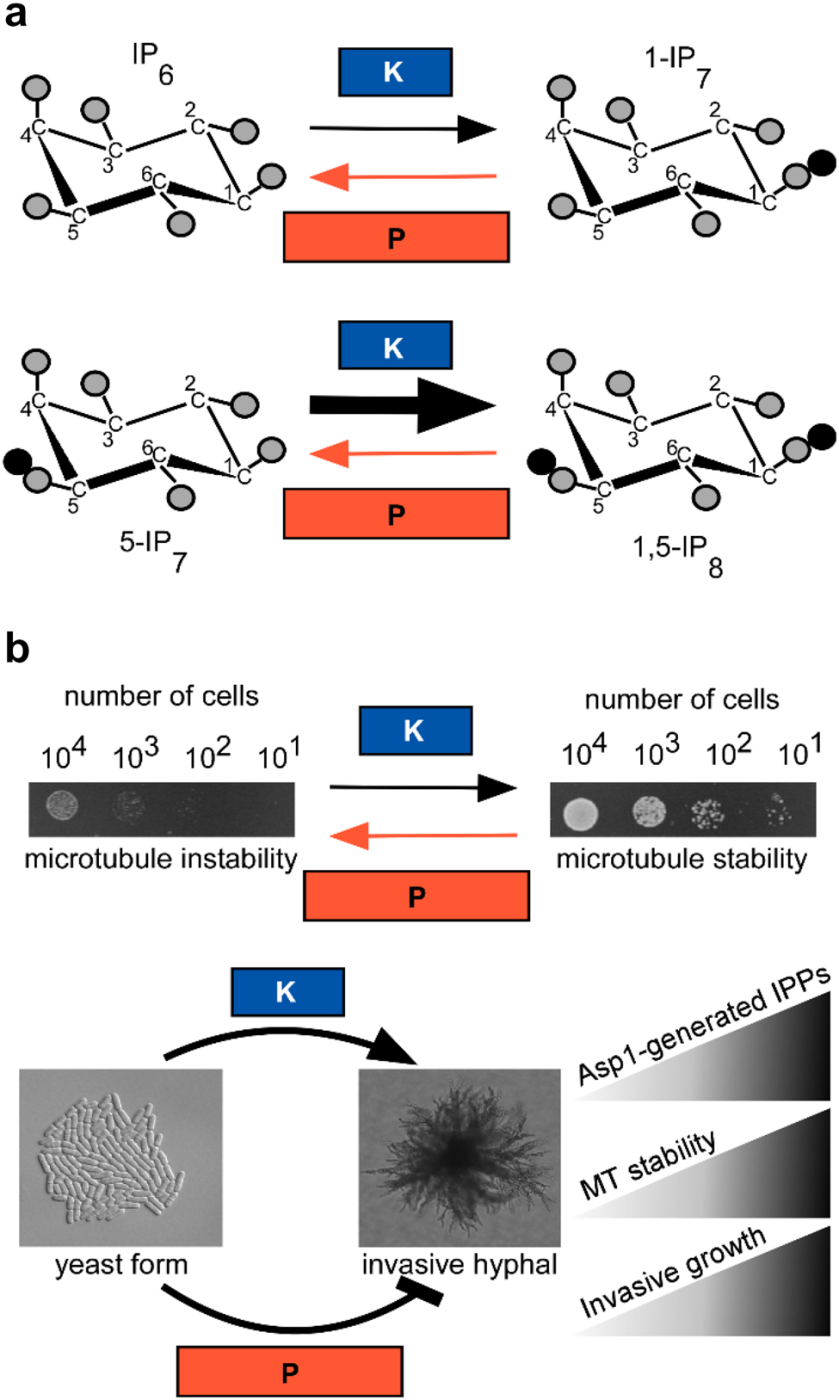
Enzymatic activity and biological function of the kinase (K) and pyrophosphatase (P) domain of Asp1. **a** Schematic representation of the reactions catalyzed by the N-terminal kinase and the C-terminal pyrophosphatase domains of *S. pombe* Asp1 [23]. The phosphate groups are shown as gray circles and the diphosphate groups are shown as gray and black circles. The reaction monitored in our in vitro assay is shown in the top panel, while the major reaction carried out in vivo is shown in the bottom panel. The thick arrow indicates that this reaction is predominant in the cells. **b** Biological function of the bifunctional Asp1 protein in *S. pombe*: Asp1 kinase generates specific IPPs, which are the substrates of the Asp1 pyrophosphatase. Increasing levels of intracellular 1,5-IP_8_ influence microtubule (MT) stability, which leads to resistance of the cells to TBZ. The top panel shows serial dilution patch tests of yeast cells growing with (left) or without (right) the MT destabilizing drug TBZ. The bottom panel shows the switch from surface, single-celled yeast growth (yeast form) to invasive pseudohyphal growth. Asp1 kinase activity is required for this switch, while Asp1 pyrophosphatase activity has an inhibitory effect [20]. The wedges on the right side of the bottom panel indicate the direct correlation of the IPP levels with the MT stability and invasive growth

The lack of a well-known characteristic Cys pattern and the sensitivity to oxygen are the reasons why the Fe–S cluster bound to the C-terminal pyrophosphatase domain of Asp1 from *S. pombe* has not been discovered until recently [13]. Asp1 belongs to the highly conserved Vip1/PPIP5Ks family, which generates a unique subclass of the soluble inositol phosphates (IPs), namely the inositol pyrophosphates (IPPs) (Fig. 1a) [14, 15]. Vip1/PPIP5Ks family members are bifunctional enzymes with an N-terminal kinase domain and a C-terminal domain with specific inositol pyrophosphate 1-phosphatase activity [16–20]. Extensive research has defined numerous biological processes controlled by inositol pyrophosphates, and in the fission yeast *Schizosaccharomyces pombe*, these signaling molecules control cell morphogenesis, microtubule stability, chromosome transmission fidelity, modulation of the actin cytoskeleton, and vacuole integrity [16, 20–24]. Specifically, as inositol pyrophosphates generated by Asp1 regulate microtubule stability, yeast cell sensitivity or resistance to microtubule poisons such as thiabendazole (TBZ) is directly correlated to lower or higher inositol pyrophosphate levels, respectively (Fig. 1b, top panel) [20, 22, 23]. The intracellular level of the inositol pyrophosphate 1,5-IP_8_ is regulated by the enzymatic activity of the Asp1 pyrophosphatase domain [23]. Thus, increased sensitivity to TBZ is a read-out for Asp1 pyrophosphatase activity, while resistance to TBZ is a read-out for an inactive Asp1 pyrophosphatase. Similarly, the transition from the normal surface single-celled growth form to the invasive hyphal growth form is controlled by 1,5-IP_8_, and a direct correlation exits between the number of invasively growing colonies and intracellular 1,5-IP_8_ levels (Fig. 1b, bottom panel) [21].

A recent study with the Asp1 pyrophosphatase variant Asp1^371−920^ showed that the recombinantly produced and aerobically purified protein initially contained substoichiometric amounts of iron and acid-labile sulfide, which were subsequently increased by chemical reconstitution [13]. The authors proposed that Asp1^371−920^ binds a [2Fe-2S]^2+^ cluster, which inhibits its pyrophosphatase activity. Surprisingly, removal of the Fe–S cluster from the reconstituted protein using sodium dithionite (DTH) and ethylenediaminetetraacetic acid (EDTA) did not restore the pyrophosphatase activity. They presented evidence that Asp1 can bind a cluster in vitro; however, whether Asp1 contains an Fe–S cluster in vivo and its biological relevance remained unclear.

In this study, we demonstrate the presence of the [2Fe-2S]^2+^ cluster in the Asp1 pyrophosphatase domain in vivo. We show that the C-terminal pyrophosphatase domain of *S. pombe* Asp1 (Asp1^365−920^) isolated from *E. coli* BL21(DE3) *∆iscR* or *S. pombe* cells under strictly anaerobic conditions contains a [2Fe-2S] cluster. We have optimized the conditions for the maturation of Asp1^365−920^ to obtain the fully assembled Fe–S cluster-containing protein without requiring subsequent chemical reconstitution. We have then characterized the structure and function of the Fe–S cluster in the as-isolated Asp1^365−920^ protein using electronic absorption spectroscopy (EAS), X-ray absorption spectroscopy (XAS), and electron paramagnetic resonance (EPR) spectroscopy. Additionally, we have purified Asp1^365−920^ under aerobic conditions, resulting in apo protein, and maturated the protein using ^57^Fe to prepare samples for Mössbauer spectroscopy. However, our data show that chemical reconstitution yields an inhomogeneous sample. To identify potential ligands of the cofactor, we have exchanged each of the four cysteines 607, 663, 864, and 879 to serine by site-directed mutagenesis and, in addition, generated a quadruple mutant (QM) with these four cysteines replaced by serine. Furthermore, we have investigated the function of Asp1^365−920^ in the presence or absence of the [2Fe-2S]^2+^ cluster using in vivo and in vitro activity assays.

## Materials and methods

### Chemicals and buffers

All chemicals were of analytical grade or better. Buffers were prepared using Milli-Q-water.

### Strains and plasmids

All strains used are listed in Supplementary Table 1 and all plasmids used are listed in Supplementary Table 2. Generation of the pKM36-*asp1*^365−920^ construct was performed as previously described [20]. Plasmids harboring *asp1*^365−920,C607S^, *asp1*^365−920,C663S^, *asp1*^365−920,C879S^, *asp1*^365−920,C864S^, and *asp1*^365−920,C607S,C663S,C864S,C879S^ are derivatives of pKM36. The variants were generated by directed mutagenesis using a QuikChange II site-directed mutagenesis kit (Agilent Technologies) and cloned into the *E. coli* expression vector pKM36 (a gift from Dr. K. Mölleken, Heinrich-Heine-Universität, Düsseldorf, Germany) to generate GST-tagged proteins via homologous recombination in *S. cerevisiae* [25]*.* The expression constructs pJR2-3XL-*asp1*, pJR2-3XL-*asp1*^D333A^, and pJR2-3XL-*asp1*^365−920^ for expression in *S. pombe* were generated as previously described [21]. The plasmids pJR2-3XL-*asp1*^C607S^, pJR2-3XL-*asp1*^C663S^, pJR2-3XL-*asp1*^C864S^, and pJR2-3XL-*asp1*^C879S^ were generated according to the same protocol. In brief, PCR fragments were generated by directed mutagenesis using a QuikChange II site-directed mutagenesis kit (Agilent Technologies) and cloned into pJR2-3XL [26] via homologous recombination in *S. cerevisiae* [25]. The quadruple mutant pJR2-3XL-*asp1*^365−920, C607S, C663S, C864S, C879S^ was obtained by digestion of pJR2-3XL with the restriction enzymes NotI and PstI (New England Biolabs) and homologous recombination in *S. cerevisiae* [25], followed by leucine selection. For constructing the pJR2-3XL-*asp1*^365−920^-GST plasmid, the GST fragment was generated by PCR and cloned into the vector pJR2-3XL-*asp1*^365−920aa^-GFP [21], which was previously digested using the restriction enzymes SmaI and NcoI (New England Biolabs) via homologous recombination in *S. cerevisiae* [25], followed by leucine selection.

### Anaerobic gene expression in *E. coli*, cell harvest, and lysis

Overnight starting cultures of *E. coli* BL21(DE3), *E. coli* BL21(DE3) pISC, *E. coli* BL21(DE3) pSUF containing the plasmid pKM36-asp1^365−920^, and *E. coli* BL21(DE3) ∆*iscR* containing either the plasmid pKM36-*asp1*^365−920^ or one of the corresponding plasmids harboring the gene encoding one of the Asp1^365−920^ variants were used to inoculate Terrific Broth (TB) medium at 1% (v/v). TB medium was supplemented with kanamycin (50 µg/ml), ampicillin (100 µg/ml), and ferric ammonium citrate (2 mM final concentration). Cells were cultivated aerobically at 37 °C and 160 rpm until the optical density measured at 600 nm (OD_600_) reached 2. For anaerobic cell growth, cultures were then moved to an anaerobic chamber (Coy Laboratory Products, Grass Lake, MI, USA) containing 98% N_2_ and 2% H_2_. Gene expression was induced by adding 0.5 mM isopropyl β-d-1-thiogalactopyranoside (IPTG). To facilitate Fe–S cluster assembly and anaerobic metabolism, 2 mM *L*-cysteine as well as 25 mM sodium fumarate were added. Cultures were stirred on a magnetic stirrer at room temperature (RT) for 20 h following induction. Cells were harvested for 10 min at 6000×*g* and 4 °C under argon atmosphere to maintain anaerobic conditions. For cell lysis, cells were resuspended in 25 mM Tris–HCl pH 8.0, 150 mM NaCl. EDTA-free cOmplete™ Protease Inhibitor Cocktail Tablets (Roche, Basel, Switzerland) were added as needed. After stirring at RT for 20 min under anaerobic conditions, the suspension was sonicated (Bandelin electronic, Berlin, Germany) for 20 min with an amplitude of 60% and a pulse of 1 s every 3 s using a VS70/T sonotrode under argon atmosphere. Lysates were clarified by centrifugation at 40000×*g* under argon atmosphere.

### Aerobic gene expression of Asp1^365−920^ in *E. coli*

To obtain a higher protein yield, necessary for Mössbauer spectroscopy, expression of *asp1*^365−920^ was carried out under aerobic conditions resulting in a higher cell density and expression level. Aerobic expression was essentially the same as anaerobic expression, but the cultures were not transferred to an anaerobic chamber prior to induction and were incubated at 25 °C and 160 rpm for 20 h. Cell harvest and lysis were performed as previously described, but without exchanging the atmosphere with argon. Isolation of the protein under aerobic conditions results in apo protein, which was used for activity assays as well as for reconstitution experiments.

### Aerobic gene expression in *S. pombe*, cell harvest, and lysis

*S. pombe* transformants, expressing *asp1*^*365−920*^ on the pJR2-3XL plasmid via the *nmt1*^+^ promoter, were grown in minimal medium with supplements for 18 h at 30 °C. Then, 2 × 10^8^ cells of a logarithmic *S. pombe* culture were harvested by centrifugation for 5 min at 3500 rpm. The pellet was washed with 5 ml STOP buffer (0.9% NaCl, 1 mM NaN_3_, 10 mM EDTA, and 50 mM NaF). Cells were resuspended under anaerobic conditions in 500 µl HB15 buffer (25 mM 4-Morpholinepropanesulfonic acid, 60 mM *β*-glycerophosphate, 15 mM *p*-nitrophenylphosphate, 15 mM MgCl_2_, 15 mM ethylene glycol-bis(β-aminoethyl ether)-*N*,*N*,*N′*,*N′*-tetraacetic acid, 1 mM 1,4-dithio-D-threitol (DTT), 0.1 mM sodium orthovanadate, 1% Triton X-100, 1 mM phenylmethanesulfonyl fluoride, and cOmplete™ Protease Inhibitor Cocktail Tablet, Roche, Basel, Switzerland) and lysed using glass beads. Then, 500 µl of HB15 buffer was added, and samples were purged with argon gas, and centrifuged for 30 min at 4 °C and 11,000×*g*. The pelleted cells were then washed with HB15 buffer and centrifuged for 30 min at 11,000×*g*.

### Anaerobic protein isolation

Asp1 purifications were carried out under strictly anaerobic conditions at RT. Lysate was applied to a GSTrap HP column (GE Healthcare, Little Chalfont, UK) with a bed volume of 5 ml equilibrated with 25 mM Tris–HCl pH 8.0 and 150 mM NaCl using an ÄKTAprime plus system (GE Healthcare, Little Chalfont, UK). The column was then washed with 10 column volumes of 25 mM Tris–HCl pH 8.0, 150 mM NaCl before the target protein was eluted with the same buffer containing 10 mM L-glutathione. Fractions containing the brownish target protein were pooled. The protein was transferred into 50 mM HEPES, pH 7.5; 150 mM KCl using a HiTrap desalting column (GE Healthcare, Little Chalfont, UK). Protein concentrations were determined using the Bradford method [27]. The protein yield from cell cultures cultivated under anaerobic conditions was 0.006 µmol per l cell culture and from cell cultures cultivated under aerobic conditions 0.01 µmol per l cell culture.

### Chemical reconstitution of Asp1^365−920^ variants

100 µM of aerobically isolated Asp1^365−920^ was incubated on ice with 50 mM DTT under anaerobic conditions for 1 h and then supplemented with 400 µM ferric ammonium citrate, 400 µM ferrous ammonium sulfate, and 800 µM Li_2_S. After 1 h, the protein was applied to a HiTrap Desalting column (GE Healthcare, Little Chalfont, UK) to separate the protein from excess iron and sulfide. For Mössbauer spectroscopy, aerobically purified Asp1^365−920^ was reconstituted following the described protocol but with ^57^FeCl_3_ instead of ferrous ammonium sulfate and ferric ammonium citrate.

### In vitro activity assay of Asp1^365−920^

Protein in the apo form, isolated under aerobic conditions, and in the Fe–S cluster bound form, isolated under anaerobic conditions, was used for the measurements of the enzymatic activity in vitro. For the kinase reaction, 1 µg of purified Asp1^1−364^ protein was incubated for 16 h at 37 °C with 300 µM inositol hexakisphosphate (IP_6_) (Sigma-Aldrich, St. Louis, Missouri, USA), 5 mM ATP, 6 mM phosphocreatine, 2.5 µl creatine phosphokinase (500 U/ml) in 30 mM HEPES pH 6.8, 50 mM NaCl, 6 mM MgSO_4_, and 1 mM DTT in a total volume of 50 µl, followed by Asp1^1−364^ inactivation at 65 °C for 20 min. The Asp1^1−364^ inactivation was verified by performing a kinase assay with the treated Asp1^1−364^ protein. For the phosphatase assay, 30 µl of the generated 5-diphosphoinositol (1,2,3,4,6) pentakisphosphate (1-IP_7_) were incubated with 2 µg of Asp1^365−920^ for 3 h or 18 h at 37 °C in a total volume of 50 µl. The assay was carried out under anaerobic conditions. The samples were analyzed on a 35% polyacrylamide gel using electrophoresis, followed by staining with Toluidine Blue O (Merck KGaA, Darmstadt, Germany) to visualize the inositol compounds.

### Invasive-growth assay

Invasive-growth assays were carried out as described [21]. *S. pombe asp1*^*H397A*^ transformants were pre-grown in minimal medium without thiamine to allow high expression of the plasmid-encoded *asp1* variants via the thiamine-repressible promoter *nmt1*^+^. 10^4^ cells were then patched on plasmid-selective minimal medium agar plates and incubated for 21 days at 25 °C. Plates were washed thoroughly to eliminate all surface grown yeast, followed by microscopic determination of the number of invasively growing colonies. Plates were photographed using Axiovert 40FL, Axiocam HR, and AxioVisionLE64 programs.

### Determination of metal content

10 µM freshly and anaerobically isolated proteins were precipitated with 3% trace-metal grade nitric acid before analysis. The protein was pelleted by centrifugation for 20 min at 15,000×*g*. The supernatant was then transferred to a metal-free centrifugation tube (VWR, Radnor, PA, USA). The Fe content of the protein was determined by inductively coupled plasma mass spectroscopy (ICP-MS) using an Agilent 7500ce ICP-MS instrument (Agilent Technologies, Ratingen, Germany) in the Central Institute for Engineering, Electronics and Analytics (ZEA-3) at Forschungszentrum Jülich. Samples were measured in triplicates.

### Electronic absorption spectroscopy

EAS was used to determine the iron–sulfur cluster content of Asp1^365−920^, Asp1^365−920,C607S^, Asp1^365−920,C663S^, Asp1^365−920,C864S^, Asp1^365−920,C879S^, and Asp1^365−920,C607S,C663S,C864S,C879S^. Electronic absorption spectra were recorded using a Cary-60 spectrophotometer (Agilent Technologies, Ratingen, Germany) with 1 nm bandwidth, a scanning speed of 120 nm/min, and a 1 cm path-length quartz cuvette at RT. For the Fe–S cluster stability assays, we recorded a spectrum of the protein isolated under anaerobic conditions at time point 0. After the first measurement, the sample was purged with air for a few minutes using a pipette and the cuvette was stored without a lid until the next measurement. This procedure was repeated after each measurement.

### X-ray absorption spectroscopy

Samples of Asp1^365−920^ isolated from *E. coli* BL21(DE3) *∆iscR* under anaerobic conditions were loaded into custom Delrin X-ray sample cells with a Kapton tape window, frozen and stored in liquid nitrogen until measurement. The total protein concentration of the measured sample was 0.35 mM for a total Fe concentration of 0.7 mM.

Fe K-edge XAS data were recorded on SSRL beamline 9–3 using a 100-element solid-state Ge detector (Canberra) with an SPEAR storage ring current of ∼500 mA at a power of 3.0 GeV as previously described [28]. The incoming X-rays were selected using a Si(220) double-crystal monochromator and an Rh-coated mirror was utilized for harmonic rejection. Samples were maintained at ~ 10 K in a liquid helium flow cryostat. Data were calibrated by simultaneously measuring an iron foil, with the first inflection point set to 7111.2 eV.

Assessment of short XANES scans (~ 2–10 min) was used to assess radiation damage and determine dwell time limits. When necessary, the incident beam was attenuated by detuning and/or insertion of aluminum foil into the beam path at varying attenuation lengths. Only scans that showed no evidence of radiation damage were included in the final analysis.

Individual partial fluorescence yield (PFY)-XAS scans were evaluated and processed in Matlab 2017a to average selected channels of the multi-element detector and perform normalization of the averaged PFY signal by the incident beam character (I0). Final averaged scans (8 scans total) were then further processed within Athena [29], where a second-order polynomial was fit to the pre-edge region and subtracted throughout the entire EXAFS spectrum. The XAS pre-edge region was fit to pseudo-Voigt lineshape through the subtraction of the edge and the integrated area is multiplied by a factor of 100 following procedures previously described [30, 31]. A three-region cubic spline (with the AUTOBK function within Athena) was employed to model the background function to *k* = 12 Å^−1^. Fourier transforms were performed over a windowed *k*-range of 2 to 11.75 Å^−1^ and presented without a phase shift correction.

Theoretical EXAFS spectra were calculated using Artemis utilizing the multiple scattering FEFF6 code [29]. The EXAFS amplitude, χ(*k*), is given by:

$$\chi (k) = \sum\limits_{R} {S_{0}^{2} N\frac{{f_{{{\text{eff}}}} (k)}}{{kR^{2} }}\sin (2kR + \phi_{k} )e^{{ - 2kR/\lambda_{k} }} e^{{ - 2\sigma^{2} k^{2} }} } ,$$

where ${S}_{0}^{2}$ is the overall many-body amplitude factor, *N*, is the degeneracy of the paths, |*f*_eff_(*k*)| is the effective scattering amplitude, and *R* is the absorber-scatterer distance. A Debye–Waller like factor, exp(− 2σ^2^*k*^2^) is also included to account for disorder. Finally, *λ*_*k*_ is the mean free path of the photoelectron and *ϕ*_*k*_ is the total photoelectron wave phase shift for the interaction between the absorber and the scatterer.

The Fourier-transform spectrum of each was fit over a range of *R* = 1.0–3.0 Å (non-phase shift corrected). The FT is the product of a transform of the *k*^3^-weighted EXAFS spectrum with a Hann window over the range of *k* = 2–11.75 Å^−1^. By grouping similar scattering paths of a common coordination shell and increasing its degeneracy, *N*, the number of variables used for that coordination shell is minimal, two variables: *σ*^2^ and Δ*R*. A single Δ*E*_0_ variable is used for all paths in a given fit for a shift from the set *E*_0_ of 7118.5 eV of the EXAFS spline. *S*_0_^2^ was set to 0.9 for all paths. Goodness of final fits were evaluated by their reduced *χ*^2^ value, defined below and calculated by Artemis:

$${\chi }^{2}=\frac{{N}_{idp}}{{\epsilon }^{2}{N}_{fit}}\sum_{i}^{M}{\left({d}_{i}-{f}_{i}\right)}^{2}.$$

Here, *d* is the raw data, f is the fitted data, and *ϵ* is the estimated noise level, summed over *M* points of the spectrum. The reduced *χ*^*2*^ value is normalized for the number of variables used (*N*_*idp*_/*N*_*fit*_), so that fits of differing number of paths may be statistically compared.

### Mössbauer spectroscopy

Samples for Mössbauer spectroscopy require the incorporation of the isotope ^57^Fe into the Asp1^365−920^ protein, and thus, the protein was isolated aerobically in the apo form and maturated using chemical reconstitution as described above. Mössbauer spectra were recorded on a conventional spectrometer with alternating constant acceleration cooled with an Oxford Instruments Variox cryostat, using a ^57^Co/Rh (1.8 GBq) *γ*-source. Samples were measured at 80 K with no applied magnetic field. Isomer shifts are relative to iron metal at 300 K. Spectra were simulated and fitted using Lorentzian quadrupole doublets using the in-house software MFIT (developed by Eckhard Bill).

## Results

### Expression in BL21(DE3) ***∆iscR*** cells leads to Fe–S cluster-containing Asp1^365−920^

In our previous work, we have characterized several recombinant Asp1^365−920^ variants with electronic absorption spectroscopy and the spectra show no or very low bands at 410 nm, which would indicate the presence of a Fe–S cluster [23]. All genes were expressed in *E. coli* Rosetta(DE3) cells and the resulting proteins were isolated in the presence of atmospheric oxygen. High-level expression in conventional cell strains, including *E. coli* Rosetta (DE3), leads to partially loaded Fe–S proteins, because the iron–sulfur cluster (ISC) assembly machinery cannot meet the great demand of Fe–S clusters. In addition, protein isolation and purification under aerobic conditions leads to degradation of the oxygen-sensitive Fe–S clusters. Notably, the degree of sensitivity of Fe–S clusters can vary from protein to protein, and thus, some Fe–S proteins may not degrade when handled under aerobic conditions [32]. Therefore, we compared different protocols for producing Fe–S proteins to determine if we can isolate Asp1^365−920^ in the cluster bound form. Hereby, we expressed the truncated version of the *asp1* gene encoding the residues 365–920 in different cell strains, including *E. coli* BL21(DE3) and BL21(DE3) *∆iscR* cells, which was genetically engineered to increase the amount of iron–sulfur cluster-containing protein significantly [33]. The deletion of the gene encoding the negative regulator protein of the *isc* operon (IscR) leads to an elevated level of the ISC proteins responsible for Fe–S biosynthesis and the cells are capable of producing a higher amount of Fe–S cluster-containing proteins. We also used protein isolated from BL21(DE3) cells and performed chemical and semi-enzymatic reconstitution reactions using the IscS and *L*-cysteine as a sulfur source in the enzymatic reactions. Furthermore, we analyzed protein obtained from BL21(DE3) cells containing an additional plasmid encoding either the ISC or SUF machinery. The resulting proteins were isolated in an anaerobic chamber with an atmosphere of 96% nitrogen and 4% hydrogen gas and analyzed using EAS, as well as ICP-MS.

**Fig. 2:**
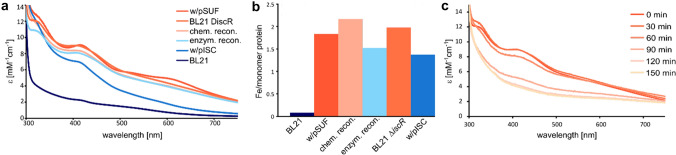
Asp1^365−920^ isolated from different cell strains compared with samples maturated by reconstitution. **a** Electronic spectra of the protein samples isolated from BL21(DE3) containing the plasmid pACYC-Duet-1-*suf* (dark orange); BL21(DE3) *∆iscR* (orange); BL21(DE3) followed by chemical reconstitution (light orange); BL21(DE3) followed by semi-enzymatic reconstitution (light blue); BL21(DE3) containing the plasmid pACYC184*iscS-fdx* (blue); and BL21(DE3) (dark blue). **b** Iron content of the same samples as in (**a**) determined by inductively coupled plasma mass spectroscopy. **c** Electronic spectra of Asp1^365−920^ exposed to air

Our data show that Asp1^365−920^ contains a [2Fe-2S] cluster, when isolated under anaerobic conditions from cell strains tailored for Fe–S protein production. Electronic spectra of Asp1^365−920^ (Fig. 2a) produced in BL21(DE3) cells suggest that the majority of the protein is present in the apo form. Coexpression in cells harboring the pACYC184*iscS-fdx* [34] (pISC) or pACYC-Duet-1-*suf* [6] (pSUF) plasmid results in protein with a high Fe–S cluster content, indicated by the bands at 325 nm, 410 nm, and 470 nm. S-to-Fe charge transfer bands in this range of the spectrum are characteristic for Fe–S clusters [35, 36]. The features observed at wavelengths higher than 500 nm are not present in every sample and may be a result of Fe–S aggregates that co-purify with the protein. The protein isolated from BL21(DE3) *∆iscR* cells also shows intense bands in the spectrum, suggesting that the majority of Asp1^365−920^ contains an Fe–S cluster. Protein isolated from BL21(DE3) in the apo form and then reconstituted using either an inorganic or biological source of sulfur show similar levels of Fe–S protein, which are slightly lower than the proteins from the BL21(DE3) *∆iscR* or BL21(DE3) pSUF cells.

The molar extinction coefficients (*ε*) at 410 nm for all samples except the protein isolated from BL21(DE3) cells are between 7 and 10 mM^−1^ cm^−1^, which is in good agreement with values found in the literature of ~ 1–10 mM^−1^ cm^−1^ [37]. Interestingly, protein production in BL21(DE3) *∆iscR* cells results in a higher fraction of Fe–S-containing protein than in BL21(DE3) pISC cells, although the same proteins should be upregulated. A possible explanation could be that the additional plasmid in the cells has a negative effect on protein production or that the levels of ISC proteins vary between the two cell strains. To quantify the amount of iron that is bound to Asp1^365−920^, we analyzed the samples that were used for EAS with ICP-MS. This technique can be used to measure the iron content in the samples and the protein content can be determined using the Bradford method [27]. These two values can then be used to calculate the number of iron ions per monomer. Our results show that protein produced in BL21(DE3) cells and isolated under anaerobic conditions contains almost no Fe–S cluster as the iron value is close to 0 (Fig. 2b). The other protocols lead to values close to two irons per monomer, which supports the presence of a [2Fe-2S] cluster in Asp1^365−920^ and suggest that the fraction of holo protein is close to 100%. Chemical reconstitution results in a value that is above two iron per monomer, indicating some unspecific iron binding. In agreement with the spectroscopic results, protein production in BL21(DE3) *∆iscR* and BL21(DE3) pSUF cell strains results in proteins samples with fully assembled [2Fe-2S] clusters. In this study, we have used the BL21(DE3) *∆iscR* cells for producing Asp1^365−920^ for the majority of experiments, because the co-expression with another plasmid implies the risk of the bacteria removing the additional plasmid.

### Asp1^365−920^ isolated from the native organism also contains an Fe–S cluster

Chemical reconstitution of proteins has previously been reported to result in artefacts. The unspecific incorporation of iron–sulfur clusters inside cells has not been reported previously and is unlikely due to the high specificity of the assembly machinery. However, we aimed at validating that the inorganic cofactor is also incorporated into the Asp1^365−920^ protein in its native organism, *S. pombe*. The *asp1*^365−920−GST^ fusion gene was expressed in *S. pombe,* and the resulting protein was isolated and purified under strictly anaerobic conditions. The electronic spectrum (Supplementary Fig. 1) confirms the presence of the Fe–S cluster, although with an extinction coefficient at 410 nm that is considerably lower than for Asp1^365−920^ isolated from the BL21(DE3) strains tailored for Fe–S protein production. The decreased extinction coefficient of the protein isolated from *S. pombe* indicates that only a part of the protein binds the inorganic cofactor, which is most likely a result of the high levels of expression and the inability of the endogenous Fe–S cluster assembly machinery to incorporate the cofactor under these conditions. The presence of the [2Fe-2S]^2+^ cluster in Asp1^365−920^ isolated from the native organism further supports the biological relevance of the inorganic cofactor for its function.

### The Fe–S cluster in Asp1^365−920^ is redox inactive

The capability to switch between different oxidation states is a crucial feature for proteins that are involved in electron transfer or redox chemistry. The potential of Fe–S clusters to take part in redox reactions can be estimated by incubating the Fe–S protein with different amounts of reducing agents, such as DTH. A previous study suggested the presence of an oxidized [2Fe-2S]^2+^ cluster in Asp1^371–920^ [13]. This type of cluster is EPR silent. Reduced [2Fe-2S]^+^ clusters with four cysteine ligands typically exhibit average *g* values in the range 1.94 to *g* = 1.96 [36, 38]. In the previous study, reconstituted Asp1^371–920^ protein was used and no EPR signal characteristic of a *S* = 1/2 [2Fe-2S]^+^ was observed [13]. However, when samples were reduced anaerobically with a twofold excess of DTH in the EPR tube, they detected weak rhombic EPR signals with an average *g* value of 1.96 that accounted for 0.06 spins/Asp1^371−920^. Our attempts to reduce the in vivo prepared samples with one and two equivalents of DTH were not successful, as shown by the EPR spectra of Asp1^365−920^ with and without two equivalents of DTH (Supplementary Fig. 2). Neither spectrum shows evidence for any reduced [2Fe-2S]^+^ cluster as previously observed. Our samples did exhibit a sharp signal at *g* = 4.3 characteristic of high-spin mononuclear ferric iron that is estimated to be no more than 10% of the sample. Furthermore, a weak but sharp isotropic signal at *g* ~ 2 is also observed with relaxation properties similar to an organic radical. Both the high-spin ferric iron and radical-like signal may arise as a result of cluster decomposition. Notably, the length of the pyrophosphatase domain was slightly shorter than the one used in this study with residues 365–370 missing; however, the potential cluster ligands are not located in this region, and we therefore expect no differences in cluster coordination.

To further investigate the potential reduction of the cluster in Asp1^365−920^, we incubated samples with different amounts of DTH under anaerobic conditions. Reduction of a [2Fe-2S]^2+^ cluster to [2Fe-2S]^+^ may be detected by a decrease of the absorbance at 410 nm and 470 nm and simultaneous increase at 400 nm and 550 nm [39]. The electronic spectra of Asp1^365−920^ do not show any changes upon treatment with one equivalent of reducing agent (Supplementary Fig. 3), indicating that the oxidation state of the cluster did not change, in agreement with the EPR observations. Further addition of reducing equivalents led to loss of the characteristic absorption bands at 410 nm and 470 nm, suggesting decomposition of the Fe–S cluster. In summary, our electronic absorption spectra and the lack of a characteristic signal for the reduced form of the cluster are in agreement with the presence of a [2Fe-2S]^2+^ cluster in the as-isolated Asp1^365−920^ protein, as previously proposed. However, our results suggest that the cluster is redox inactive.

### The Fe–S cluster degrades when exposed to oxygen

Most iron–sulfur clusters are damaged or degraded when exposed to oxygen, and therefore, protein isolation and handling are usually performed under anaerobic conditions. [2Fe-2S] cluster-containing proteins with four cysteine residues as coordinating ligands are particularly stable and an excellent example for a highly oxygen-stable Fe–S protein is *E. coli* [2Fe-2S]-ferredoxin [32]. This Fe–S protein can be isolated and even crystallized under aerobic conditions and still contains a fully occupied [2Fe-2S] cluster [40]. The behavior of Fe–S clusters in the presence of oxygen can provide insights into the function of the cofactor. Degradation of the cluster in the presence of oxygen may potentially indicate a role in oxidative stress response. In *E. coli*, the [2Fe-2S] transcriptional factor, SoxR, functions as a sensor of oxidative stress [41]. Modulation of the Fe–S cluster in SoxR was proposed to control the protein’s function in transcription.

To analyze the stability of the [2Fe-2S]^2+^ cluster in Asp1^365−920^ in the presence of oxygen, we exposed the anaerobically isolated protein samples to air and monitored the Fe–S cluster degradation utilizing electronic absorption spectroscopy. Our results show that the cluster in Asp1^365−920^ is stable in an aerobic environment for approximately 30 min (Fig. 2c). After 60 min, a small fraction of the cluster in Asp1^365−920^ is degraded, and after 120 min, most of the cluster is not present anymore. We have also monitored the cluster degradation of Asp1^365−920^ protein isolated from *S. pombe* induced by exposure to oxygen and we observe similar changes in the electronic spectrum as for protein isolated from *E. coli* BL21(DE3) *∆iscR* (Supplementary Fig. 4).

### Characterization of the Fe–S cluster in Asp1 using X-ray absorption spectroscopy

**Fig. 3:**
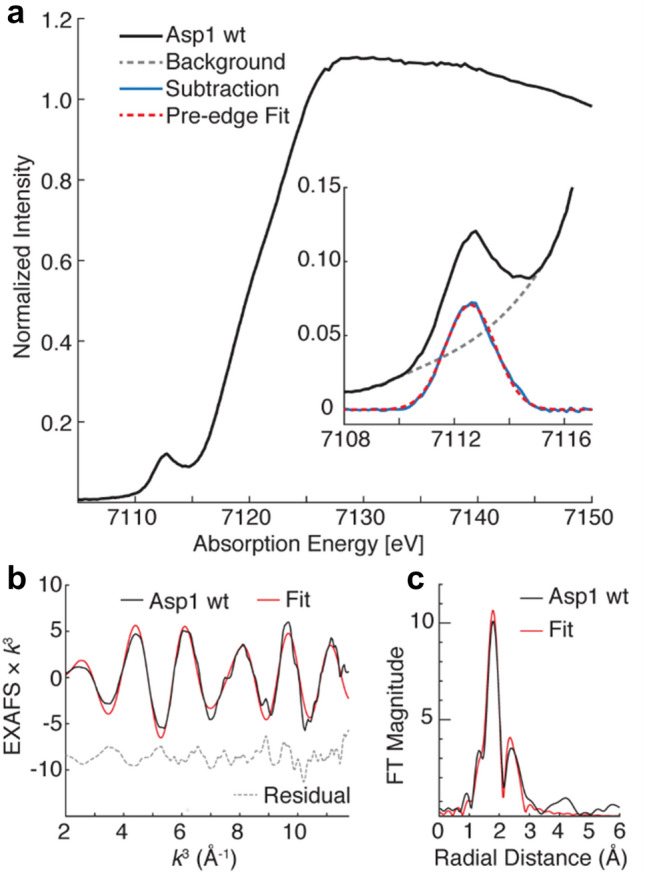
Spectroscopic characterization of wild-type Asp1^365−920^ (Asp1 wt). **a** Fe K-edge X-ray absorption spectrum of Asp1^365−920^ with the pre-edge region expanded within the inset. A cubic spline fitting of the edge and background (gray dashed line) is subtracted from the pre-edge feature (blue) and fitted with a single pseudo-Voigt lineshape (red dashed line). The fitted pre-edge feature is centered at 7112.6 eV, a full-width half-max of 1.82 eV and 0.146 units of normalized intensity. **b** The *k*^3^-weighted EXAFS of Asp1^365−920^ (black) with fitted EXAFS (red) and residual (gray dashed) over a *k*-range of 2–11.75 Å^−1^. **c** The non-phase shifted Fourier transform of the *k*^3^-weighted EXAFS is displayed (black) with a respective fit (red)

To obtain more information about the electronic structure and the coordinating ligands of the Fe–S cluster, we performed X-ray absorption spectroscopy, including extended X-ray absorption fine structure (EXAFS) analysis. The Fe K-edge XAS spectrum of the as-isolated Asp1^365−920^ (Fig. 3a) exhibits a rising-edge of ~ 7120 eV and a single pre-edge feature at 7112.6 eV of 14.6 units of intensity. The pre-edge energy is in good agreement with ferric iron and established [2Fe-2S]^2+^ clusters (Supplementary Fig. 5) [42–44]. The white line of Asp1^365−920^ appears sharper and more intense than what is typically observed for tetrathiolate-coordinated irons [42]. Additionally, the pre-edge has a lower intensity than commonly observed for other Fe–S biological sites of local iron tetrahedral symmetry. This may indicate light-atom coordination to the ferric iron, or the presence of a small amount of exogenous octahedrally coordinated ferric iron species which would possess a less intense pre-edge.

The *k*^3^-weighted EXAFS (Fig. 3b) is dominated by an Fe–S scattering interaction as indicated by the intense, partly uniform sinusoidal oscillation in a *k*-range of 2–7 Å^−1^ and an Fe–Fe contribution at higher *k*, yielding a deconstructive interference at approximately *k* ~ 8 Å^−1^, characteristic of Fe–S clusters. The non-phase shifted Fourier transform of the EXAFS spectrum (Fig. 3c) yields an intense radial shell at *R* ~ 1.8 Å, and a second less intense shell at *R* ~ 2.4 Å. The first and second radial shells are the Fe–S and Fe–Fe scattering interactions, respectively. The intensity ratio of the two shells is typical of [2Fe-2S] clusters, whereas high-nuclearity clusters, such as a typical [4Fe-4S] cluster, would possess more intense Fe–Fe scattering interactions relative to the Fe–S scattering interaction (Supplementary Fig. 13).

The EXAFS of Asp1^365−920^ is well fitted by an *N* = 4 Fe–S scattering interaction at a mean scattering distance of 2.25 Å and a Debye–Waller like disorder value of *σ*^2^ = 6.72 × 10^–3^ Å^2^ (Supplementary Table 3). The fitted fourfold degenerate Fe–S scattering path is in good agreement with typical Fe–S(cys) and Fe-(µ-S^2−^) distances found in oxidized ferredoxin clusters [43]. The Fe–Fe scattering interaction is fit to a distance of 2.73 Å by a well-ordered scattering interaction, which is reflected by the *σ*^2^-value of 3.57 × 10^–3^ Å^2^. Attempts to further refine the fitting of the EXAFS data by inclusion of an additional Fe–N/O scattering interaction of shorter distance were not conclusive (Supplementary Fig. 6, Supplementary Table 3). Inclusion of another Fe–N/O scattering interaction yielded fits with better goodness of fit values; however, other parameters of the fit are not physically intuitive, including either negative *σ*^2^ values or no support of cysteine ligation in contradiction of the mutagenesis results, discussed below.

As the technique is a bulk probe and reports the average iron spectrum, influence from minor iron species may also contribute. While the assignment of the first coordination shell is not definitive from the EXAFS spectroscopy, the formation of a Fe-S cluster is clearly observed by the unmistakable Fe–Fe interaction. It is therefore concluded that the cluster is formed in vivo and a [2Fe-2S] cluster is best supported by the EXAFS analysis. The most important finding is that this cluster is formed without the need for reconstitution with iron and sulfur.

We attempted Mössbauer spectroscopy measurements of Asp1^365−920^ protein obtained by aerobic purification and chemical reconstitution using ^57^FeCl_3_. Electronic absorption spectroscopy (Supplementary Fig. 7) revealed very low cluster content in the aerobically purified Asp1 (extinction coefficient is 2.09 mM^−1^ cm^−1^ at 410 nm), while the ^57^Fe-reconstituted Mössbauer sample had a spectrum (extinction coefficient of 8.37 mM^−1^ cm^−1^ at 410 nm) almost identical to the Fe–S bound form (extinction coefficient of 8.98 mM^−1^ cm^−1^ at 410 nm). Despite this, the spectrum revealed an inhomogeneous sample whose components could not unambiguously be assigned to either a [2Fe-2S] or [4Fe-4S] cluster (Supplementary Fig. 8 and Supplementary Discussion).

### Cysteine residues 607, 663, 864, and 879 are involved in binding the Fe–S cluster

After characterizing the structure and coordination environment of the [2Fe-2S]^2+^ cluster in the Asp1^365−920^ protein, we aimed at identifying the residues involved in linking the inorganic cofactor to the protein backbone. Fe–S clusters are predominantly ligated by cysteine residues; however, they can also be coordinated by histidine, aspartate, arginine, serine, tyrosine, or glutamate residues [36]. Several consensus sequence motifs are known for [2Fe-2S] cluster coordination, typically involving four cysteine residues as ligands, yet it remains challenging to identify the coordinating residues based on the sequence. To pinpoint the residues involved in cluster coordination, we performed site-directed mutagenesis and characterized the ability of Asp1^365−920^ to incorporate a [2Fe-2S] cluster.

**Fig. 4:**
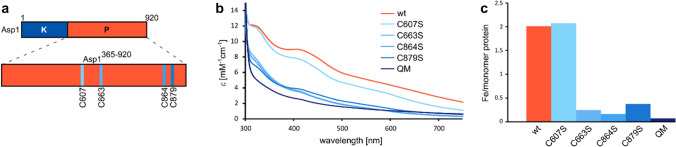
Characterization of binding and stability of the [2Fe-2S] cluster in the Asp1^365−920^ variants. **a** Schematic representation of the Asp1^365−920^ phosphatase domain used for this study in orange with the location of the four cysteines that were replaced utilizing site-directed mutagenesis highlighted in blue. **b** Electronic spectra of Asp1^365−920^ wt in orange and the variants C607S, C663S, C864S, C879S, and QM (different shades of blue) as-isolated from *E. coli* BL21(DE3) *∆iscR.* **c** Iron content of the proteins shown in (**b**) determined with ICP-MS

It was previously described that the exchange of the cysteine residues 607, 663, 864, 868, 879, and 905 leads to a reduced iron content of Asp1^371−920^ [13]. Three of these cysteines, 663, 864, and 879, are highly conserved in some fungal homologs of Asp1. Cysteine 868 in Asp1 aligns with a highly conserved aspartate residue, which could be a potential oxygenic ligand for an Fe–S cluster. As our spectroscopic data suggest that the cluster is coordinated by cysteines, we concluded that this Cys is a potential ligand of the cofactor. Cysteine 905 in Asp1 is not conserved in other yeasts and is, therefore, most likely not involved in cluster ligation. To pinpoint the residues coordinating the cluster, we generated and produced five new Asp1^365−920^ variants: C607S, C663S, C864S, C879S, and the quadruple mutant C607S C663S C864S C879S (QM). The position of these cysteine residues in Asp1 is shown in Fig. 4a. All gene constructs were expressed in *E. coli* BL21(DE3) *∆iscR* and the Asp1^365−920^ variant proteins were isolated under strictly anaerobic conditions, followed by the analysis of the Fe–S cluster content by electronic absorption spectroscopy and inductively coupled plasma mass spectroscopy (Fig. 4b, c). Our data show that the Asp1^365−920^ QM is not capable of binding a significant amount of Fe–S cluster and the metal content is lower than 0.1 iron per monomer. The three Asp1^365−920^ variants C663S, C864S, and C879S contain a very low amount of cluster indicated by the band intensities in the electronic spectra and the low metal content of 0.1–0.4 iron per monomer. While the metal content of the Asp1^365−920^ C607S protein is the same as for the wild-type protein with 2.0 iron per monomer, a comparison of the molar extinction coefficients at 410 nm indicates that the amount of Fe–S cluster is approximately 20% lower in the C607 variant. Taken together, our results demonstrate that the cysteines 663, 864, and 879 are essential for binding the [2Fe-2S] cluster to the protein backbone. Cysteine 607 seems to have an impact on the cluster, because the extinction coefficient at 410 nm is lower compared to Asp1^365−920^; however, the role of this residue in cluster coordination is not clear.

To better understand the role of C607, we also investigated the behavior of the Asp1^365−920^ C607S variant towards oxygen. When exposing the C607 variant to air, the cluster starts to degrade within 30 min and the cluster is not detectable after 120 min (Supplementary Fig. 9). A comparison of the oxygen sensitivity of Asp1^365−920^ wild-type and C607S (Supplementary Fig. 10) suggests that the [2Fe-2S] cluster in Asp1^365−920^ C607S is more sensitive to oxidative degradation, which points towards Cys 607 being a ligand for the Fe–S cluster. Furthermore, we have also treated the Asp1^365−920^ C607S variant with DTH under anaerobic conditions and the electronic spectra (Supplementary Fig. 11) reveal a similar behavior of the cluster as in wild-type Asp1^365−920^. The addition of two or more equivalents of reductant leads to a decrease of the absorption band at 410 nm without the emergence of another signal that would indicate a reduction of the [2Fe-2S]^2+^ cluster to [2Fe-2S]^+^. These data suggest that the Fe–S cluster decomposes upon reduction.

To ensure that the cluster content of all Asp1^365−920^ variants is not a result of accidental degradation during the isolation and purification of the protein, we performed chemical reconstitution on all Asp1^365−920^ variants. The electronic spectra of the proteins after the reconstitution reaction and buffer exchange (Supplementary Fig. 12) show that the values for the molar extinction coefficients of wild-type Asp1^365−920^ (11.91 mM^−1^ cm^−1^ at 325 nm and 8.98 mM^−1^ cm^−1^ at 410 nm) and C607S (11.64 mM^−1^ cm^−1^ at 325 nm and 7.76 mM^−1^ cm^−1^ at 410 nm) did not increase. This indicates that the Asp1^365−920^ protein was isolated with a fully assembled [2Fe-2S] cluster. It also shows that the lower amount of Fe–S cluster in the C607S variant is due to the inability of this variant to stabilize the cluster, pointing to a possible function of this Cys as a cluster ligand. Furthermore, chemical reconstitution of the Asp1^365−920^ variants C663S, C864S, C879S, and QM did not increase the signals at 325 nm and 410 nm, indicating that these mutants are not capable of binding an Fe–S cluster.

### The Fe–S cluster does not influence phosphatase activity in vitro

Previous studies suggested that the [2Fe-2S]^2+^ cluster substantially inhibits the pyrophosphatase activity of Asp1. Thus, we investigated the activity of Asp1^365−920^ in the presence and absence of the Fe–S cluster. Therefore, we produced Asp1^365−920^ under aerobic conditions, which results in the apo form, and compared the activity with the Fe–S cluster-containing protein as isolated. To measure the enzymatic activity of these Asp1^365−920^ variants, we used an in vitro test system that had allowed us previously to show for the first time that a member of the PPIP5K/Vip1 family is a bifunctional enzyme and that the hitherto C-terminal “phosphatase-like domains” of PPIP5K/Vip1 family members are indeed phosphatases [20]. The 1-pyrophosphatase activity of this family is conserved [13, 16, 45, 46].

**Fig. 5:**
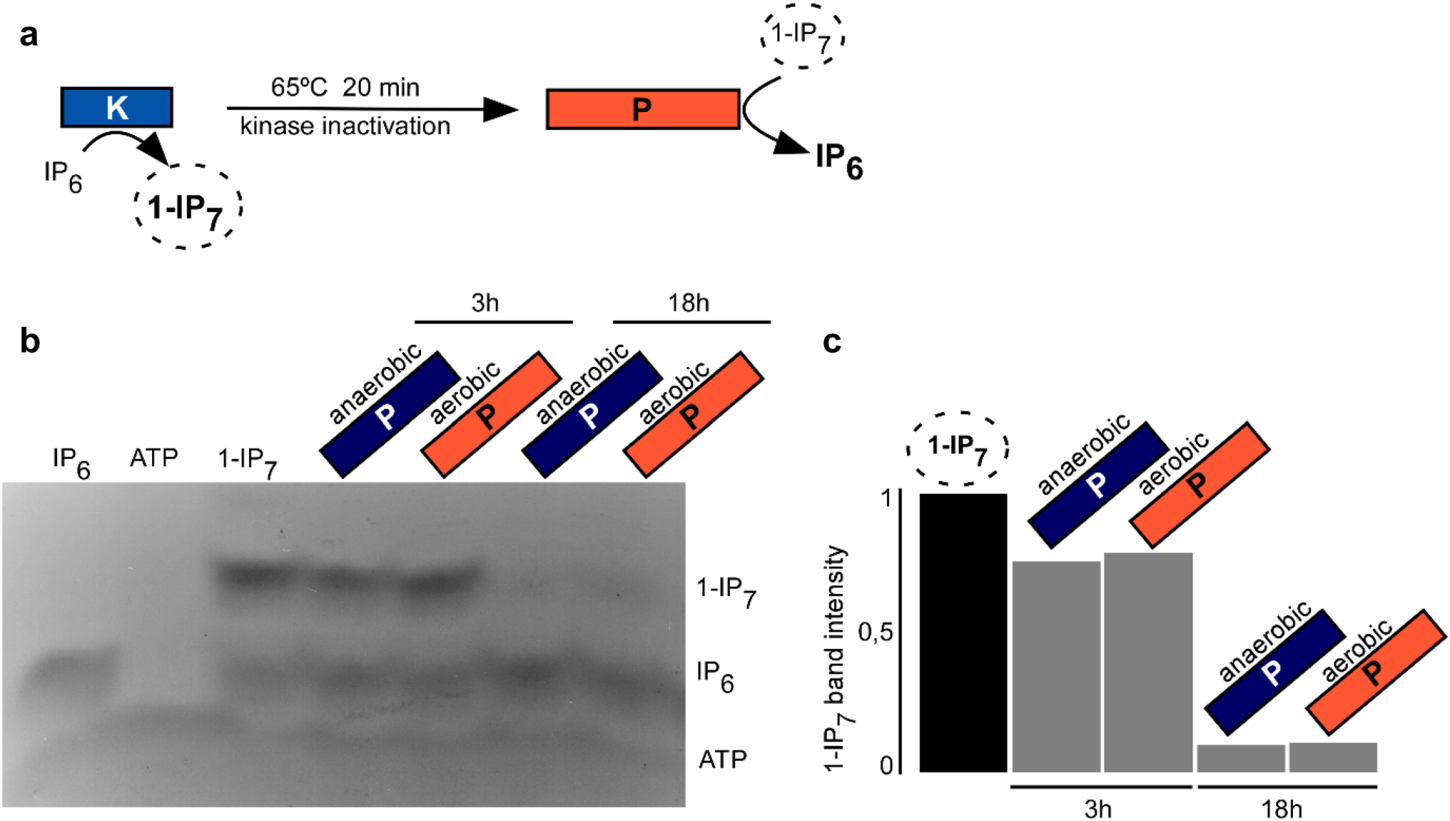
Activity of Asp1^365−920^ in vitro*.* **a** Schematic representation of the in vitro pyrophosphatase assay. **b** In vitro pyrophosphatase assay using the indicated Asp1^365−920^ variants. 2 µg of the indicated proteins were added to Asp1 kinase-generated 1-IP_7_ (input is shown in the lane labelled 1-IP_7_) and incubated for 3 h or 18 h. The resulting inositol polyphosphates were resolved on a 35.5% PAGE gel and stained with toluidine blue. The assay was performed twice with reproducible results. **c** Quantification of 1-IP_7_ bands shown in (**b**)

Incubation of Asp1^1−364^ produced 1-IP_7_ with Asp1^365−920^ in either apo or holo form led to a moderate (3 h) or massive (18 h) decrease of this inositol pyrophosphate (Fig. 5b, c). Thus, as both forms of Asp1^365−920^ showed comparable enzymatic activity, we conclude that the presence of the [2Fe-2S]^2+^ cluster does not inhibit phosphatase activity in vitro, in contrast to what has been reported in a previous study by Wang et al. [13].

### Expression of the Asp1^365−920/QM^ variant induces TBZ hypersensitivity and reduces the ability of yeast cells to grow invasively similar to Asp1^365−920^

**Fig. 6:**
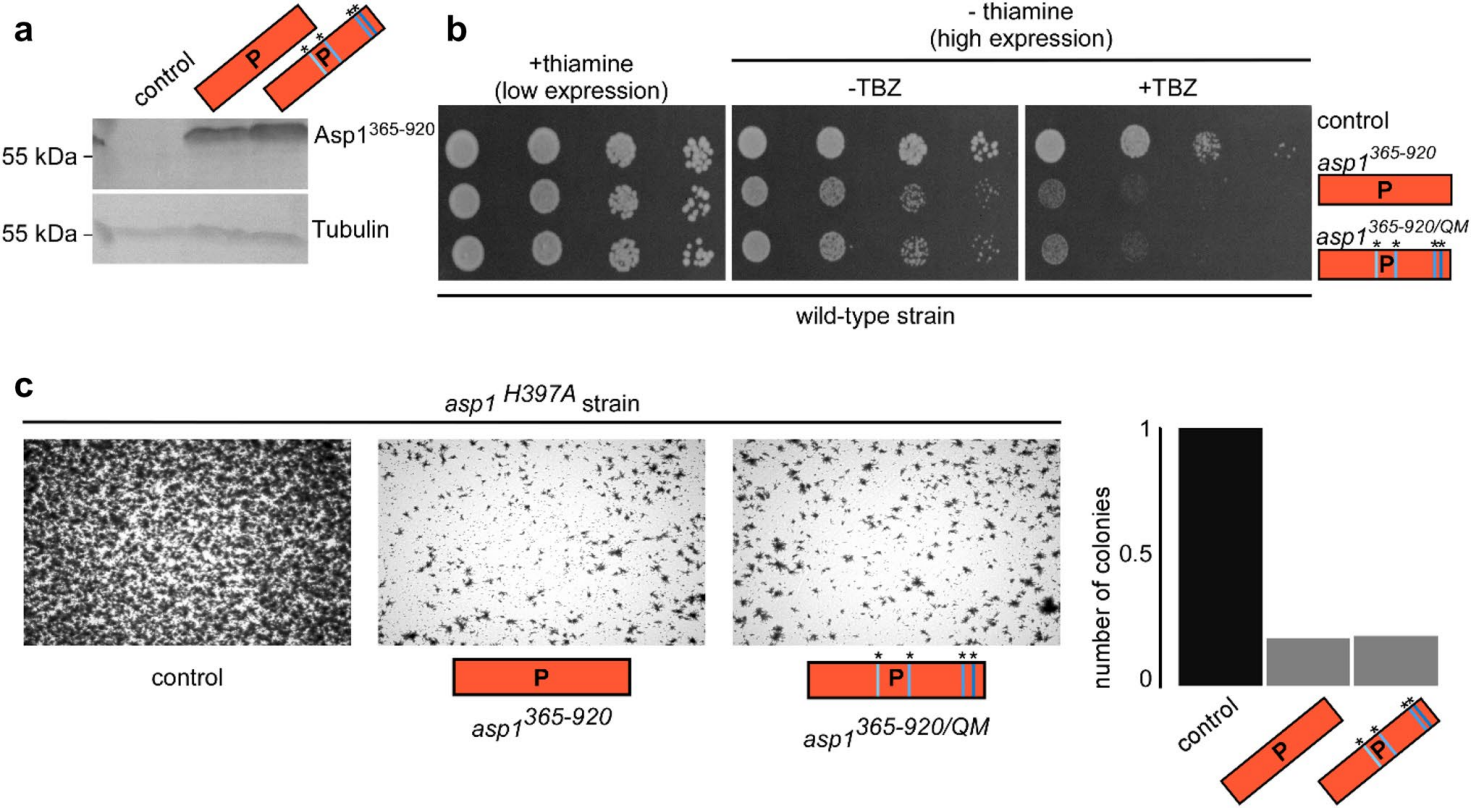
Activity of Asp1^365−920^ in vivo. **a** Western blot analysis of a wild-type strain transformed with a control plasmid or plasmids harboring the indicated *asp1* variants. Transformants were grown in thiamine-less, plasmid-selective minimal media to highly express the *asp1* variants via the *nmt1*^+^ promoter. Similar amounts of protein extracts were resolved by SDS-PAGE and probed with anti-Asp1 or anti-tubulin antibodies, respectively. **b** Serial dilution patch tests (10^4^–10^1^ cells) of the transformants shown in (**a**). Transformants were grown either in the presence or absence of thiamine, which will lead to low or high expressions from the *nm1*^+^ promoter and with or without the microtubule-destabilizing drug TBZ. **c** Invasive-growth test of strain *asp1*^*H397A*^ transformed with a control plasmid or plasmids harboring the indicated *asp1* variants. Transformants were grown in the absence of thiamine to allow high expression of *asp1*^*365−920*^ and *asp1*^*365−920/QM*^. Transformant cells were incubated for 21 d under plasmid-selective conditions, plates washed to eliminate all surface grown cells and the number of invasive colonies quantified via microscopy

Activity measurements with apo and holo protein have shown that the inorganic cofactor does not influence the ability to carry out the pyrophosphatase function in vitro. To investigate whether the presence of the Fe–S cluster affects the function of the Asp1 pyrophosphatase in vivo, we analyzed microtubule stability and the ability to form invasively growing colonies, as these two biological processes are controlled by 1,5-IP_8_ in fission yeast. As intracellular levels of 1,5-IP_8_ are controlled by the activity of the Asp1 pyrophosphatase, the in vivo activity of Asp1 pyrophosphatase variants can be determined using the following read-outs: expression of plasmid-borne functional Asp1 pyrophosphatase variants in *S. pombe* transformants will lead to (1) increased sensitivity to the microtubule-destabilizing drug TBZ and (2) a reduction in the number of cells that can switch to invasive growth. The plasmid-borne expression of either wild-type Asp1^365−920^ or the variant Asp1^365−920/QM^ via the thiamine-repressible promoter *nmt1*^+^ (Fig. 6a) both resulted in a virtually identical reduction of growth in the presence of the microtubule-destabilizing drug TBZ (Fig. 6b).

Next, we used the *asp1*^*H397A*^ strain, which expresses an endogenous Asp1 variant with an inactive pyrophosphatase [23]. Thus, this strain has significantly higher 1,5-IP_8_ levels than wild-type and, as a consequence, shows an increased ability to switch to invasive growth (Fig. 6c, leftmost panel) [21]. Expression of either wild-type Asp1^365−920^ or mutant Asp1^365−920/QM^ in this strain resulted in a decrease of invasively growing colonies (Fig. 6c, middle and right panels and quantification of the number of invasively growing colonies). Again, as shown for the previous in vivo assay, the read-out of Asp1^365−920/QM^ and Asp1^365−920^ expression is very similar. We conclude that the enzymatic activities of Asp1^365−920/QM^ and Asp1^365−920^ are comparable, and that the absence of the inorganic cofactor does not affect Asp1^365−920/QM^ function in vivo in the biological processes tested.

## Discussion

During recombinant gene expression, proteins are produced to an abnormally and excessively high level, which frequently leads to incomplete Fe–S cluster incorporation. Several approaches have been used to increase the Fe–S cluster content in proteins, including the co-expression with plasmids containing the *isc* or *suf* operons or the deletion of the regulating *iscR* gene. Expression of the *asp1* gene variant in these cell strains yields high amounts of Fe–S cluster-containing Asp1^365−920^ protein, supporting the biological relevance of the inorganic cofactor. Despite the absence of the SUF machinery in all yeast strains, including *S. pombe*, the co-expression with the *suf* operon results in high protein yields. Because the ISC and SUF machinery are almost completely interchangeable in bacteria, both machineries are supposedly capable of interacting with the same proteins. Therefore, it is not surprising that the SUF machinery is efficiently incorporating Fe–S clusters into a eukaryotic protein.

Qualitative and quantitative analyses of Asp1^365−920^ in its Fe–S bound form using electronic absorption spectroscopy and ICP-MS, respectively, have shown that expression in BL21(DE3) *∆iscR* cells results in a high yield of Fe–S protein (Fig. 1). Chemical or semi-enzymatic reconstitution seem to result in protein with similar spectral properties and iron content. However, the iron/monomer ratios of the samples obtained by chemical reconstitution are above two, revealing unspecific iron binding. In vivo incorporation of Fe–S clusters result in samples with higher homogeneity and purity, because (1) the protein is folded correctly when the Fe–S cofactor is incorporated during or directly after translation, (2) metal-binding sites may be occupied by non-native metals such as Zn or Ni during expression or purification, leading to inaccessible metal-binding sites, (3) excess Fe–S aggregates can be formed during reconstitution that are quite difficult to separate from the protein, (4) reconstitution may result in a cluster form that is not identical to the native cluster form, and (5) adventitious iron may bind to the protein in an unspecific manner, interfering with spectroscopic characterization.

Spectral features in electronic spectra are not as pronounced as we would expect for a [2Fe-2S] cluster. For rhombic dinuclear clusters, we expect two well-resolved bands at 410 and 470 nm. The degree of which structures can be resolved decreases with increasing numbers of iron atoms; thus, we only observe one broad feature in the range of 400–450 nm for [4Fe-4S] proteins. The electronic spectra of the Asp1^365−920^ protein as-isolated as well as chemically reconstituted protein reveal one broad feature in the range of 400–500 nm. A comparison with well-characterized [2Fe-2S] and [4Fe-4S] proteins shows that the broad peak may result from two peaks at 410 and 470 nm, which are not well resolved (Supplementary Fig. 14). Additionally, the spectrum of Asp1^365−920^ also reveals a pronounced shoulder at 330 nm, like the [2Fe-2S] protein. The spectrum of the [4Fe-4S] protein has no band at 330 nm and the absorbance beyond 450 nm is significantly lower. The features in the electronic spectrum of the Asp1^365−920^ protein resemble the well-characterized [2Fe-2S] protein; however, the presence of a [4Fe-4S] species cannot be excluded based on the spectrum. The EXAFS analysis and the metal content support the presence of the [2Fe-2S] form, which is in line with a previous work that identifies a [2Fe-2S]^2+^ cluster in Asp1.

Our mutagenesis study demonstrates that the exchange of cysteines 663, 864, and 879 by serines has a dramatic impact on cluster stability. Replacement of cysteine by serine was chosen, because serines are more likely to maintain the hydrogen-bonding network in the structure compared to Ala. Mutation of cysteine 607 to Ser also leads to a decreased Fe–S cluster content (Fig. 4c); however, while the extinction coefficients at 410 nm for the C663S, C864S, and C879S variants decrease from 8.98 mM^−1^ cm^−1^ to values ranging between 3.51 and 3.98 mM^−1^ cm^−1^, the value for the C607S variant only decreases to 7.75 mM^−1^ cm^−1^. The number of irons per monomer for the C607S variant is also close to the value for Asp1^365−920^. The hydroxyl group of S607 may be capable of stabilizing the cluster to a certain extent. In this case, the question arises why the hydroxyl group is not able to stabilize the cluster when introduced at another position. A possible explanation may be that this cysteine is more flexible than the others or that the exchange of cysteine 663, 864, and 879 leads to local changes in the hydrogen network in a fashion that the hydroxyl group is not available for cluster stabilization. As the Asp1^365−920/QM^ variant reveals a virtually identical level of phosphatase activity, it is unlikely that amino acid exchange leads to major rearrangements. Another possibility is that there is another cysteine located in close proximity to residue 607 that is capable of rearranging to stabilize the Fe–S cluster. It has previously been reported that when cysteine ligands to an Fe–S cluster are changed to alanine, nearby cysteines can substitute and form ligands to the cluster [47]. In the absence of structural information, it is difficult to predict which residue would be a potential candidate. Finally, the cluster could be coordinated by three cysteine residues, as seen in the scaffold protein ISU1 involved in Fe–S cluster biogenesis [48]. This would lead to an open coordination site that allows potential binding partners to bind to Asp1^365−920^ through a cysteine residue that occupies the free coordination site, as observed in the IscU:IscS complex [49]. EXAFS analysis shows Fe–S scattering interaction that is typical of [2Fe-2S] clusters, which could be modelled satisfactory by a majority S coordinated iron–sulfur cluster, in agreement with the electronic spectra. Our data are consistent with the [2Fe-2S] cluster coordinated to four cysteines, we, therefore, propose that cysteine 607 is a cluster ligand.

Our activity measurements in vitro and in vivo reveal that the pyrophosphatase function is not influenced by the absence of the [2Fe-2S] cluster. For the in vitro analysis, the level of activity of Asp1^365−920^ isolated under anaerobic conditions in the [2Fe-2S] cluster-containing form or under aerobic conditions in the apo form is similar, demonstrating that the cluster is not involved in this reaction. Previously, the Fe–S cluster was proposed to inhibit the pyrophosphatase activity [13]. In that study, activity could not be recovered after removal of the Fe–S cluster, which could indicate that the loss of activity derives from the presence of excess reagents from the reconstitution reaction or Fe–S aggregates that are formed during chemical reconstitution. Another possibility is that the treatment of the protein to remove the cluster affected the structural integrity leading to an inactive pyrophosphatase domain. The authors did not provide evidence for structural integrity of the samples. This raises the question of the role of the Fe–S cluster in Asp1. A function in redox catalysis or electron transfer is highly unlikely, because all attempts to reduce the cluster have failed so far. A structural role is one possibility; however, since the pyrophosphatase domain is active in the absence of the cluster, we conclude that Asp1 is folded correctly in the absence of the Fe–S cluster.

Our present in vivo analysis tested two biological processes that require 1,5-IP_8_: microtubule stability and morphogenesis. In these assays, the absence of the [2Fe-2S] cluster in Asp1 did not lead to phenotypic consequences of the yeast strains expressing such Asp1 variants, indicating that these two processes are not affected by the absence of the [2Fe-2S] cluster. What then might the functional role of the Asp1 [2Fe-2S] cluster be? We have recently found that Asp1 interacts physically with the mitochondrially localized Met10 protein and that Met10 inhibits the pyrophosphatase activity of Asp1^365−920^ in vitro [23]. The budding yeast homologue of Met10 protein, *Sc*Met10, is a Fe–S cluster-containing protein that physically interacts with Met18/Mms19, which is part of the cytosolic iron–sulfur protein assembly (CIA) machinery [50]. Our unpublished observations indicate that an Asp1 subspecies exists in fission yeast mitochondria and Asp1 mutants give rise to altered ATP levels. Altered ATP levels were also observed in human cell lines which did not express PPIP5K [46]. We, therefore, speculate that the role of the [2Fe-2S] cluster in Asp1^365−920^ may be related to a possible interaction with the protein Met10.

## Supplementary Information

Below is the link to the electronic supplementary material.

Supplementary file1 (PDF 1381 KB)
