# Supplementary material for: The Asp1 pyrophosphatase from S. pombe hosts a [2Fe-2S]2+ cluster in vivo
Source: J Biol Inorg Chem. 2021 Feb 5;26(1):93–108. doi: 10.1007/s00775-020-01840-w (PMC8038993; doi:10.1007/s00775-020-01840-w)
Supplement: Supplementary file 1 — Supplementary file1 (PDF 1381 KB) [file 775_2020_1840_MOESM1_ESM.pdf]

Supplementary Material

for

The Asp1 pyrophosphatase from *S. pombe* hosts a $[2Fe\text{-}2S]^{2+}$ cluster *in vivo*

Hannah Rosenbach[1], Eva Walla[2], George E. Cutsail III[3], James A. Birrell[3], Marina Pascual-Ortiz[4], Serena DeBeer[3], Ursula Fleig[2,*] and Ingrid Span[1,*]

Table of Contents

Supplementary Figure 1: Electronic absorption spectra of $Asp1^{365\text{-}920}$ as-isolated from *E. coli* BL21(DE3) Δ*iscR* and *S. pombe*.
Supplementary Figure 2: Electron paramagnetic resonance spectra of wild-type $Asp1^{365\text{-}920}$ as-isolated and with sodium dithionite.
Supplementary Figure 3: Electronic absorption spectra of $Asp1^{365\text{-}920}$ exposed to different equivalents of sodium dithionite.
Supplementary Figure 4: Electronic absorption spectra of $Asp1^{365\text{-}920}$ isolated from *S. pombe* and exposed to air monitored over the time course of 150 min.
Supplementary Figure 5: Comparison of XAS data collected from $Asp1^{365\text{-}920}$ (Asp1) as isolated from *E. coli* BL21(DE3) Δ*iscR* with published data.
Supplementary Figure 6: Supplemental EXAFS fits to vary the first coordination sphere.
Supplementary Figure 7: Electronic absorption spectra of $Asp1^{365\text{-}920}$ when anaerobically and aerobically isolated from *E. coli* BL21(DE3) Δ*iscR* as well as reconstituted with ^{57}Fe for Mössbauer spectroscopy.
Supplementary Figure 8: Mössbauer spectrum of $Asp1^{365\text{-}920}$ enriched with ^{57}Fe measured at 80 K without applied field.
Supplementary Discussion: Characterization of reconstituted wild-type $Asp1^{365\text{-}920}$ by Mössbauer spectroscopy
Supplementary Figure 9: Electronic absorption spectra of the $Asp1^{365\text{-}920}$ C607S variant exposed to air monitored over the time course of 150 min.
Supplementary Figure 10: Comparing the oxygen-sensitivity of $Asp1^{365\text{-}920}$ wild-type and C607S.
Supplementary Figure 11: Electronic absorption spectra of the $Asp1^{365\text{-}920}$ C607S variant exposed to different equivalents of sodium dithionite.
Supplementary Figure 12: Electronic absorption spectra of reconstituted $Asp1^{365\text{-}920}$ variants.
Supplementary Figure 13: FT-EXAFS of theoretical $[2Fe\text{-}2S](Cys)_4$ and $[4Fe\text{-}4S](Cys)_4$ clusters demonstrating the intensity ratios of the first and second radial shells.
Supplementary Figure 14: Comparison of the electronic spectrum of $Asp1^{365\text{-}920}$ with two well-characterized [2Fe-2S] and [4Fe-4S] proteins.
Supplementary Table 1: *E. coli* and *S. pombe* strains used in this study.
Supplementary Table 2: Plasmids used in this study.
Supplementary Table 3: Fitting parameters for the Mössbauer spectrum in Supplementary Figure 8.
Supplementary Table 4: Comparison of different fits for the EXAFS data collected from $Asp1^{365\text{-}920}$ as-isolated from *E. coli* BL21(DE3) Δ*iscR*.
Supplementary References

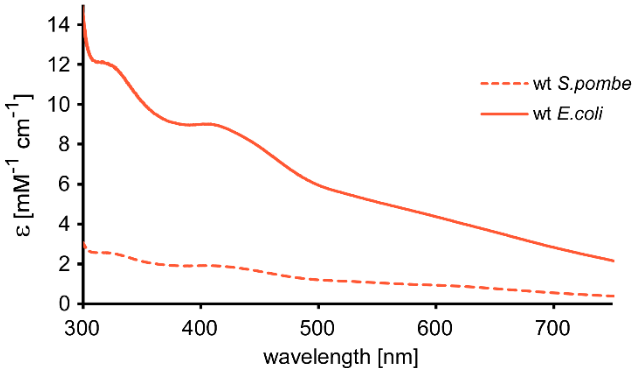

Supplementary Figure 1: Electronic absorption spectra of Asp1$^{365\text{-}920}$ as-isolated from *E. coli* BL21(DE3) Δ*iscR* and *S. pombe*.

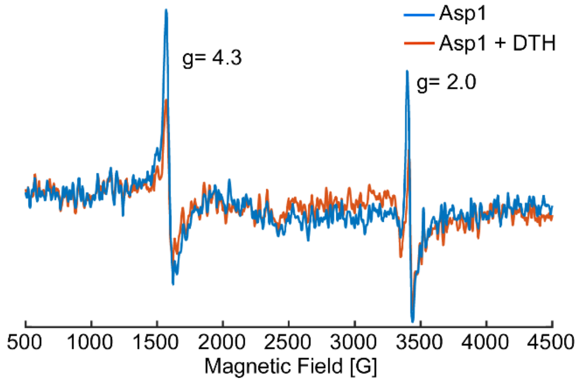

Supplementary Figure 2: Electron paramagnetic resonance spectra of wild-type Asp1$^{365\text{-}920}$ as-isolated (Asp1) shown in blue and reduced with 2 equivalents sodium dithionite (DTH) shown in orange. Spectra were collected at 10 K on Bruker E-500 spectrometer equipped with an Oxford liquid He flow cryostat. Conditions: microwave frequency, 9.63 GHz; modulation frequency, 100 kHz; modulation amplitude, 6 G; time constant, 20.48 ms; conversion time, 81.92 ms.

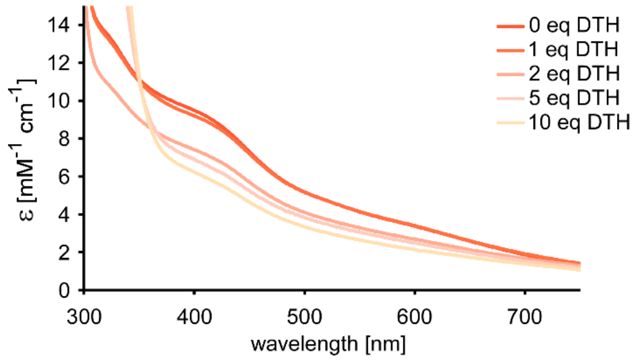

Supplementary Figure 3: Electronic absorption spectra of Asp1$^{365\text{-}920}$ exposed to different equivalents of DTH.

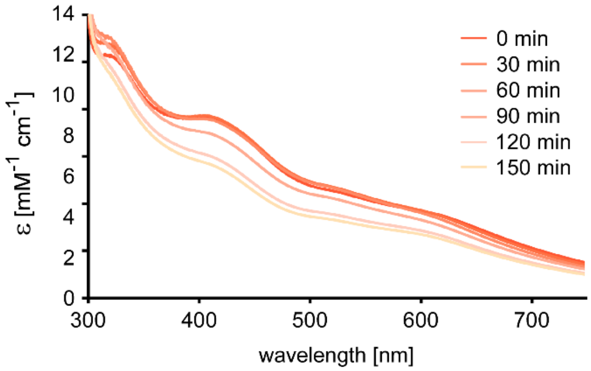

Supplementary Figure 4: Electronic absorption spectra of Asp1$^{365-920}$ isolated from *S. pombe* and exposed to air monitored over the time course of 150 min.

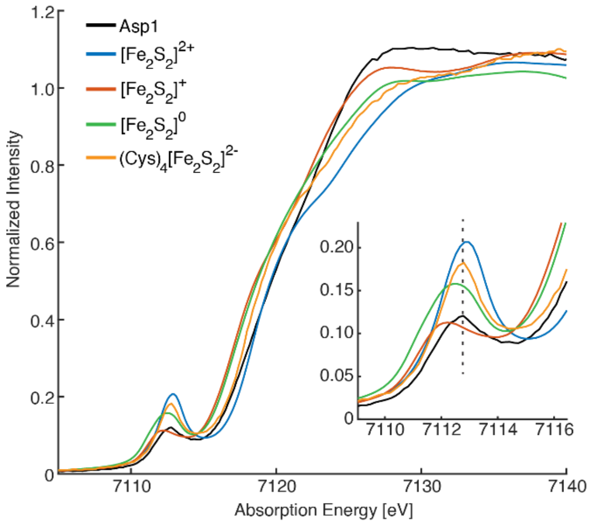

Supplementary Figure 5: Comparison of XAS data collected from Asp1$^{365-920}$ (Asp1) as isolated from *E. coli* BL21(DE3) Δ*iscR* with published data. $[Fe_2S_2]^{2+/1+/0}$ data of synthetic model complexes in the diferric, mixed valent, and diferrous states, respectively. $[L_2Fe_2S_2]^n$, where L= bis(benzimidazolato) and *n*= 2-, 3-, 4-. The data is adapted from Kowalska, J. K.; Hahn, A. W.; Albers, A.; Schiewer, C. E.; Bjornsson, R.; Lima, F. A.; Meyer, F.; DeBeer, S., "X-Ray Absorption and Emission Spectroscopic Studies of $[L_2Fe_2S_2]^n$ Model Complexes: Implications for the Experimental Evaluation of Redox States in Iron-Sulfur Clusters," *Inorg Chem* **2016**, *55* (9), 4485-4497. Copyright 2016 American Chemical Society. (https://doi.org/10.1021/acs.inorgchem.6b00295). Further permissions related to the material excerpted should be directed to the American Chemical Society.

The diferric $(Cys)_4[Fe_2S_2]^{2-}$ ferredoxin data is adapted with permission from (Foster, M. W.; Mansy, S. S.; Hwang, J.; Penner-Hahn, J. E.; Surerus, K. K.; Cowan, J. A. "A Mutant Human IscU Protein Contains a Stable [2Fe-2S](2+) Center of Possible Functional Significance," *J. Am. Chem. Soc.*, **2000**, *122* (28), 6805-6806. Copyright 2000 American Chemical Society.

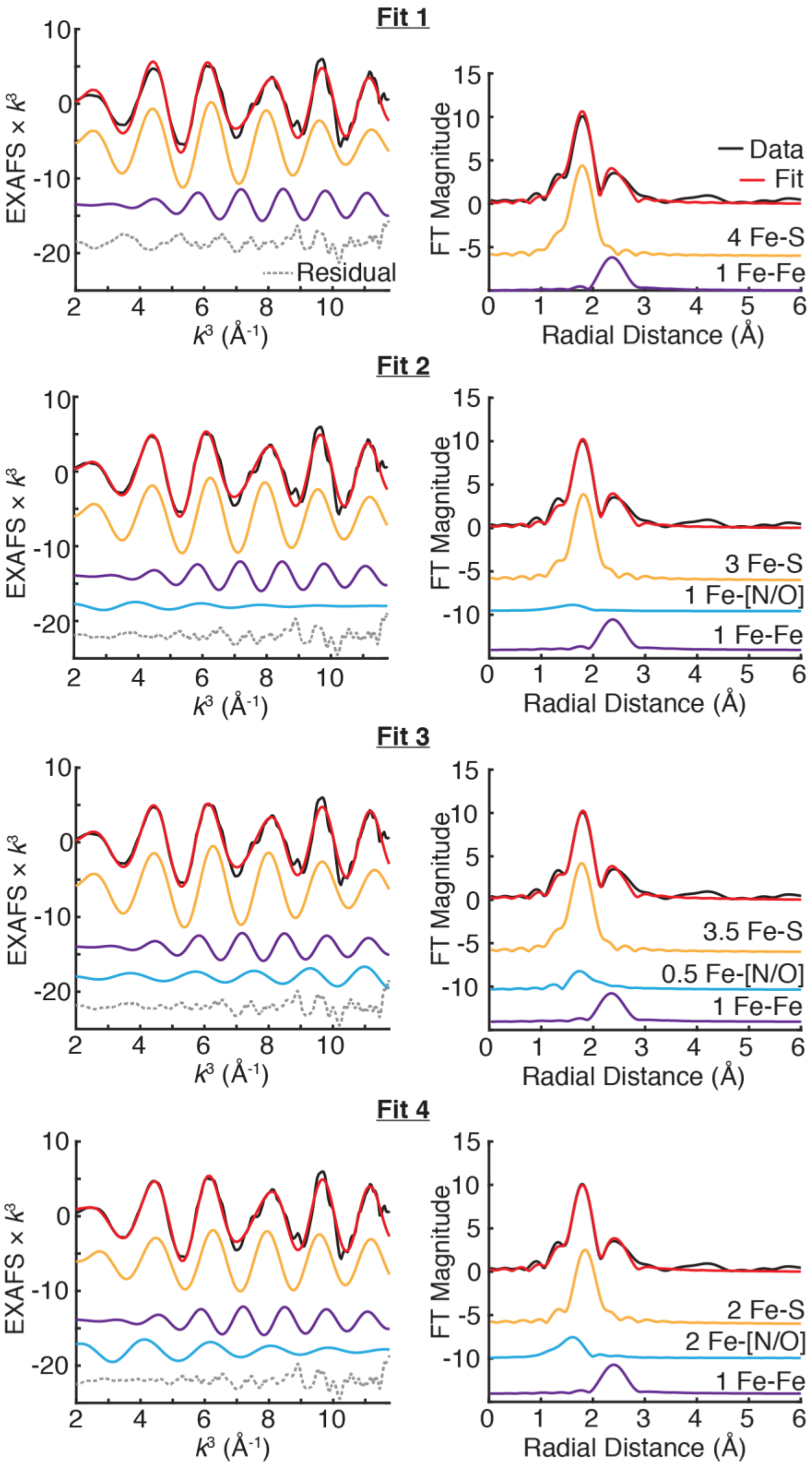

Supplementary Figure 6: Supplemental EXAFS fits to vary the first coordination sphere. Fit parameters and statistics are detailed in Supplementary Table 3.

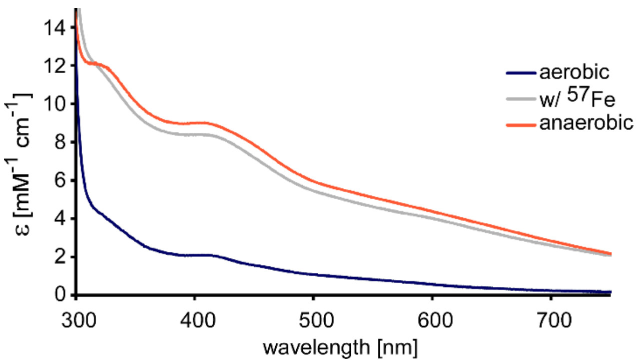

Supplementary Figure 7: Electronic absorption spectra of Asp1$^{365-920}$ when anaerobically (orange) and aerobically (blue) isolated from *E. coli* BL21(DE3) Δ*iscR* as well as reconstituted with ^{57}Fe for Mössbauer spectroscopy (grey).

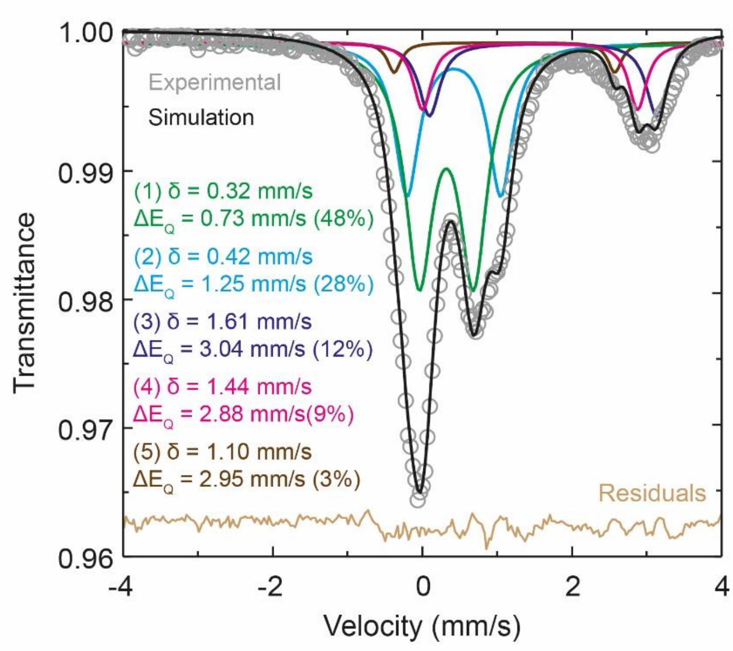

Supplementary Figure 8: Mössbauer spectrum of Asp1$^{365-920}$ enriched with ^{57}Fe measured at 80 K without applied field. The experimental spectrum (gray circles) has been fit (black line) with five components (green, blue, purple, pink and brown lines). The fitting parameters are presented in Supplementary Table 4

Supplementary Discussion: Characterization of reconstituted wild-type Asp1$^{365-920}$ by Mössbauer spectroscopy

Mössbauer spectroscopy is frequently used to obtain detailed information about the chemical environment and electronic structure of iron in Fe-S proteins. The nucleus most amenable to Mössbauer spectroscopy is ^{57}Fe, thus, protein samples have to be reconstituted or enriched with the isotope ^{57}Fe. All previous experiments have been performed with as-isolated Asp1$^{365-920}$ protein and all attempts to fully remove the inorganic cofactor led to precipitation of the protein. Therefore, we used Asp1$^{365-920}$ protein obtained by aerobic purification for chemical reconstitution using $^{57}FeCl_3$ to enrich the ^{57}Fe content. The electronic spectra (Supplementary Fig. 7) reveal that the

majority of the isolated protein is in the apo form (extinction coefficient is 2.09 $mM^{-1}cm^{-1}$ at 410 nm). The Mössbauer sample of the reconstituted Asp1 protein has an extinction coefficient of 8.37 $mM^{-1}cm^{-1}$ at 410 nm, which is almost identical to the protein isolated in the Fe-S bound form (extinction coefficient is 8.98 $mM^{-1}cm^{-1}$ at 410 nm). Thus, the protein used for Mössbauer spectroscopy has a similar Fe-S content as the protein produced in BL21(DE3) Δ*iscR* cells.

A reasonable fit to the experimental spectrum (Supplementary Figure 8) was achieved with five components (Supplementary Table 4): two quadrupole doublets with low isomer shifts (δ = 0.32 and 0.42 mm/s) and small quadrupole splittings (ΔE_Q = 0.75 and 1.25 mm/s) and three quadrupole doublets with high isomer shifts (1.10, 1.44 and 1.61 mm/s) and large quadrupole splittings (2.95, 2.88 and 3.04 mm/s). The latter are typical of high spin Fe^{2+} and are likely to be due to some iron aggregates generated during Fe-S cluster reconstitution. The isomer shift and quadrupole splitting for components 1 and 2 are more typical of iron sulfur clusters but slightly larger than those normally observed for ferric (Fe^{3+}) ions in all cysteine ligated [2Fe-2S] clusters ($\delta \approx$ 0.27 mm/s and $\Delta E_Q \approx$ 0.60 mm/s), but not large enough for ferrous (Fe^{2+}) ions in the same environment ($\delta \approx$ 0.60 mm/s and $\Delta E_Q \approx$ 2.70 mm/s), particularly as ferrous ions in iron-sulfur clusters tend to show very large quadrupole splittings [1, 2].

Interestingly, the Mössbauer parameters appear to be more in line with those of the all ferric state of His-ligated [2Fe-2S] clusters such as IscU (δ = 0.27 mm/s and ΔE_Q = 0.66 mm/s for the Cys-ligated Fe^{3+} and δ = 0.32 mm/s and ΔE_Q = 0.94 mm/s for the His-ligated Fe^{3+}) [3] or MitoNEET (δ = 0.26 mm/s and ΔE_Q = 0.47 mm/s for the Cys-ligated Fe^{3+} and δ = 0.30 mm/s and ΔE_Q = 0.96 mm/s for the His-ligated Fe^{3+}) [4], or the all ferric state of the double His-ligated Rieske [2Fe-2S] cluster (δ = 0.24 mm/s and ΔE_Q = 0.52 mm/s for the Cys-ligated Fe^{3+} and δ = 0.32 mm/s and ΔE_Q = 0.91 mm/s for the His-ligated Fe^{3+}) [5]. This demonstrates how the isomer shift and the quadrupole splitting can be affected by ligand environment around the cluster, which may explain the large isomer shifts and quadrupole splittings in Asp1. The unusual Mössbauer properties correlate with the unusual XAS spectral properties and the inability to observe an S= ½ signal in the EPR spectrum of the reduced protein.

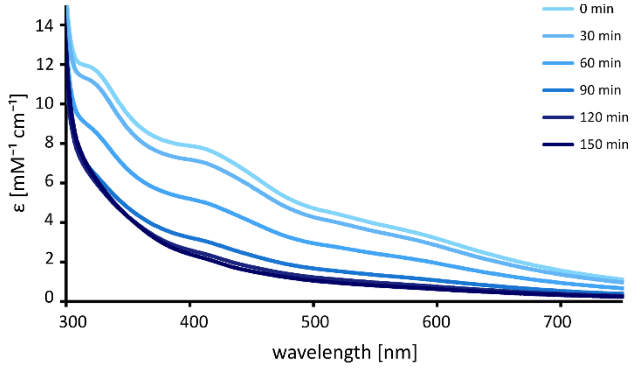

Supplementary Figure 9: Electronic absorption spectra of the Asp1$^{365\text{-}920}$ C607S variant exposed to air monitored over the time course of 150 min.

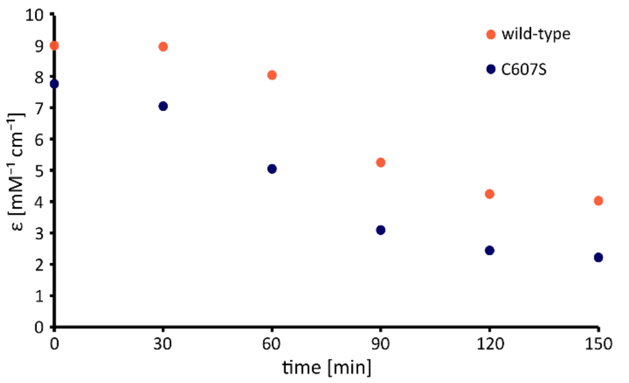

Supplementary Figure10: Comparing the oxygen-sensitivity of Asp1$^{365-920}$ wild-type (orange) and C607S (blue). The molar extinction coefficient at 410 nm is plotted against time.

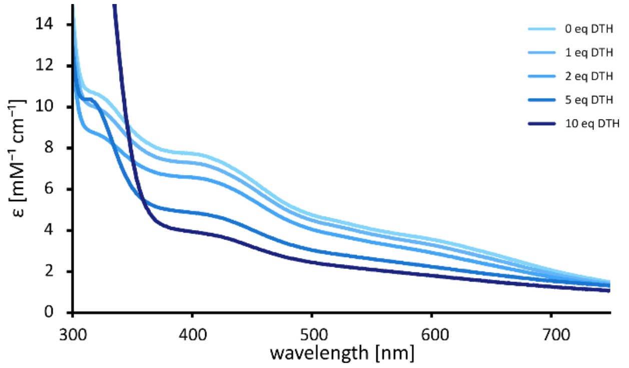

Supplementary Figure 11: Electronic absorption spectra of the Asp1$^{365-920}$ C607S variant exposed to 0 - 10 equivalents of DTH.

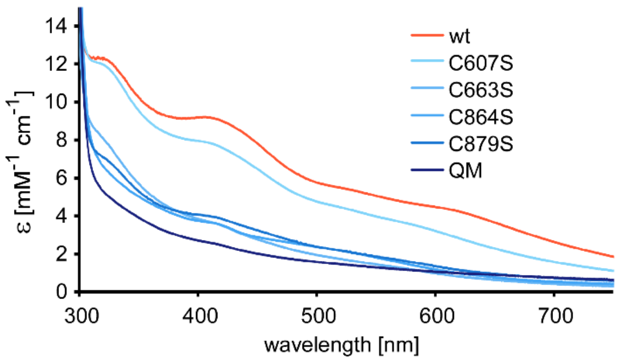

Supplementary Figure 12: Electronic absorption spectra of reconstituted Asp1$^{365-920}$ variants with the wild-type (wt) shown in orange and the variants C607S, C663S, C864S, C879S and the quadruple mutant C607S C663S C864S C879S (QM) shown in different shades of blue.

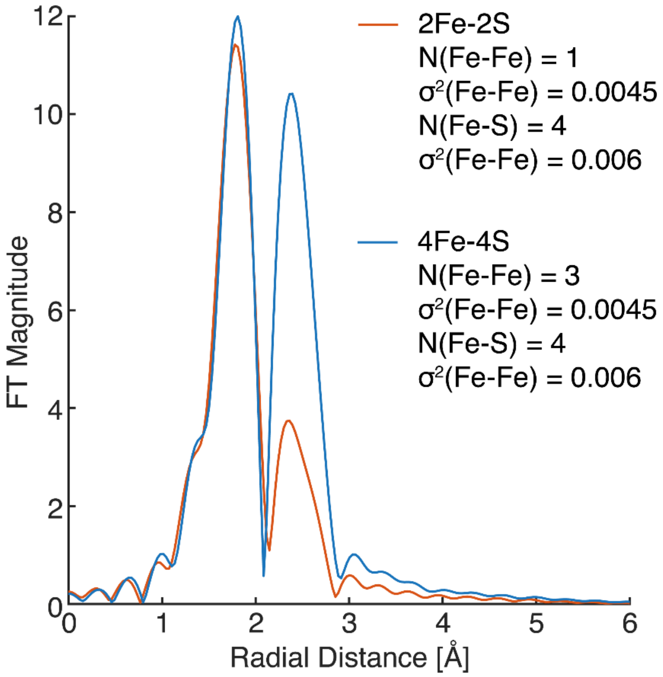

Supplementary Figure 13: FT-EXAFS of theoretical [2Fe-2S](Cys)$_4$ and [4Fe-4S](Cys)$_4$ clusters demonstrating the intensity ratios of the first and second radial shells. For each cluster, 4 degenerate Fe-S scattering interaction of ~2.25 Å are simulated and equivalent σ^2-values. The [2Fe-2S] cluster has a single Fe-Fe scattering interaction at 2.70 Å with a modeled σ^2-value of $4.5x10^{-3}$ Å^2. The [4Fe-4S] cluster has a Fe-Fe scattering path with 3-fold degeneracy at ~2.70 Å and are modeled with the same σ^2-value as the [2Fe-2S].

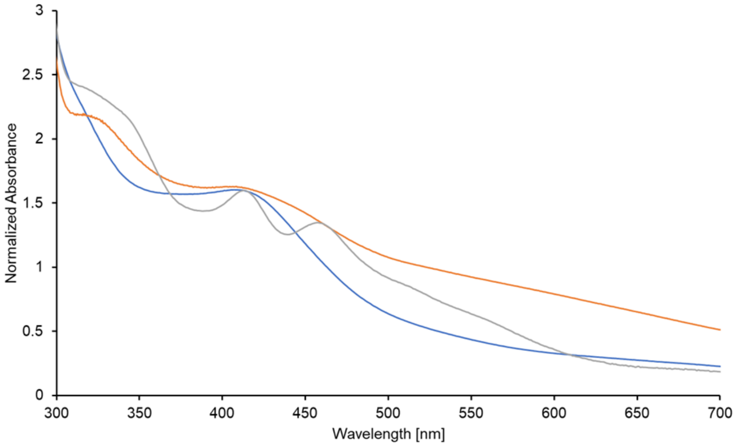

Supplementary Figure 14: Comparison of the electronic spectrum of Asp1$^{365\text{-}920}$ (orange) with two well-characterized [2Fe-2S] and [4Fe-4S] proteins. The spectrum of the [2Fe-2S]-binding Ferredoxin from *E. coli* is shown in grey and the spectrum of the [4Fe-4S]-binding HMBPP reductase from *E. coli* is shown in blue.

Supplementary Table 1: *E. coli* and *S. pombe* strains used in this study.

Name	Genotype	Reference
BL21(DE3)Δ*iscR*	F − *ompT hsdS$_B$* (r_B^- m_B^-) *gal dcm iscR::*kan (DE3)	[6]
UFY 605	his3-D1, ade6-M210, leu1-32, ura4-D18, h^-	K. Gould
UFY 1579	asp1^{H397A}::kan^R, his3-D1, ade6-M210, leu1-32, ura4-D18, h^+	U. Fleig

Supplementary Table 2: Plasmids used in this study.

For expression in *E. coli*	
pKM36-*asp1*$^{365-920}$	*Amp^R*, *tac* Promotor, *lac* Operator, *GST Factor X site*, *TRP*, based on pGEX-3X (GE Healthcare)
pKM36-*asp1*$^{365-920,C607S}$	
pKM36-*asp1*$^{365-920,C663S}$	
pKM36-*asp1*$^{365-920,C864S}$	
pKM36-*asp1*$^{365-920,C879S}$	
pKM36-*asp1*$^{365-920,C607S,C663S,C864S,C879S}$	
**For expression in *S. pombe***	
pJR2-3XL	*LEU2*, *Amp^R*, pREP3x, thiamin-repressible *nmt1*$^+$ promotor
pJR2-3XL-*asp1*	
pJR2-3XL-*asp1*$^{365-920aa}$	
pJR2-3XL-*asp1*$^{365-920aa-GST}$	
pJR2-3XL-*asp1*^{D333A}	
pJR2-3XL-*asp1*^{C607S}	
pJR2-3XL-*asp1*^{C663S}	
pJR2-3XL-*asp1*^{C864S}	
pJR2-3XL-*asp1*^{C879S}	
pJR2-3XL-*asp1*^{C607S,C663S,C864S,C879S}	
**Co-expression in *E. coli***	
pACYC-Duet-*suf* (pSUF)[22]	
pACYC*iscS-fdx* (pISC)[21]	

Supplementary Table 3: Comparison of different fits for the EXAFS data collected from Asp1$^{365\text{-}920}$ as-isolated from *E. coli* BL21(DE3) Δ*iscR*.

	N	**Path**	**R (Å)**	**σ^2 x 10^3 ($Å^2$)**	**ΔE_0 (eV)**	***F*-value**	**χ^2**
**Fit 1**	4	Fe-S	2.254	6.72	-0.883	1.437	281.63
	1	Fe-Fe	2.726	3.57			
**Fit 2**	3	Fe-S	2.258	4.73	0.989	0.876	191.16
	1	Fe-N	2.127	14.92			
	1	Fe-Fe	2.729	4.23			
**Fit 3**	3.5	Fe-S	2.238	5.79	-1.171	0.814	196.94
	0.5	Fe-N	2.163	-4.06			
	1	Fe-Fe	2.716	4.74			
**Fit 4**	2	Fe-S	2.268	2.70	3.633	0.549	150.44
	2	Fe-N	2.078	9.22			
	1	Fe-Fe	2.738	4.68			

Supplementary Table 4: Fitting parameters for the Mössbauer spectrum in Supplementary Figure 8.

Component	1	2	3	4	5
**Isomer shift (δ) [mm/s]**	0.32	0.42	1.61	1.44	1.1
**Quadrupole splitting (ΔE_Q) [mm/s]**	0.73	1.25	3.04	2.88	2.95
**FWHM [mm/s]**	0.45	0.39	0.34	0.29	0.22
**Depth [%]**	1.78	1.17	0.57	0.52	0.23
**Intensity [%]**	48	28	12	9	3
**RMSD**			0.872		

Supplementary References

1. Pandelia ME, Lanz ND, Booker SJ, Krebs C (2015) Mössbauer spectroscopy of Fe/S proteins. Biochim. Biophys. Acta - Mol. Cell Res. 1853:1395–1405. https://doi.org/10.1016/j.bbamcr.2014.12.005

2. Münck E, Debrunner PG, Tsibris JCM, Gunsalus IC (1972) Mössbauer parameters of putidaredoxin and its selenium analog. Biochemistry 11:855–863. https://doi.org/10.1021/bi00755a027

3. Chandramouli K, Unciuleac MC, Naik S, et al (2007) Formation and properties of [4Fe-4S] clusters on the IscU scaffold protein. Biochemistry 46:6804–6811. https://doi.org/10.1021/bi6026659

4. Ferecatu I, Gonçalves S, Golinelli-Cohen MP, et al (2014) The diabetes drug target MitoNEET governs a novel trafficking pathway to rebuild an Fe-S cluster into cytosolic aconitase/iron regulatory protein 1. J Biol Chem 289:28070–28086. https://doi.org/10.1074/jbc.M114.548438

5. Fee JA, Findling KL, Yoshida T, et al (1984) Purification and characterization of the Rieske iron-sulfur protein from Thermus thermophilus. Evidence for a [2Fe-2S] cluster having non-cysteine ligands. J Biol Chem 259:124–133. https://doi.org/10.1016/S0021-9258(17)43630-1

6. Akhtar MK, Jones PR (2008) Deletion of iscR stimulates recombinant clostridial Fe–Fe hydrogenase activity and H2-accumulation in Escherichia coli BL21(DE3). Appl Microbiol Biotechnol 78:853–862. https://doi.org/10.1007/s0025 3-008-1377-6
